# Supplementary material for: Cyclic Hexapeptide from Bouvardia ternifolia (Cav.) Schltdl. and Neuroprotective Effects of Root Extracts
Source: Plants (Basel). 2023 Jul 10;12(14):2600. doi: 10.3390/plants12142600 (PMC10416155; doi:10.3390/plants12142600)
Supplement: Supplementary file 1 [file plants-12-02600-s001.zip › plants-2464785-supplementary.pdf]

plants

Type of the Paper (Article)

Cyclic hexapeptide from *Bouvardia ternifolia* (Cav.) Schltdl. and neuroprotective effects of root extracts.

Yury Maritza Zapata Lopera [1,2], Gabriela Trejo-Tapia [2], Manasés González-Cortazar [1], Maribel Herrera-Ruiz [1], Alejandro Zamilpa [1] and Enrique Jiménez-Ferrer [1*]**

1 Centro de Investigación Biomédica del Sur, Instituto Mexicano del Seguro Social, Xochitepec 62790, Morelos, México
2 Centro de Desarrollo de Productos Bióticos, Instituto Politécnico Nacional, Yautepec 62730, Morelos, México
* Correspondence: enriqueferrer_mx@yahoo.com (E.J.-F.) ** Second corresponding author (G.T.-T.).

Content

Figure S1. GC–MS Analysis of the BtD extract

Figure S2. GC–MS Analysis of the BtH extract

Figure S3. Spectrum UV and HPLC of cyclic hexapeptide

Figure S4. Mass spectrum (MS) of cyclic hexapeptide

Figure S5. ^{1}H NMR (CD_3OD, 400 MHz) of cyclic hexapeptide

Figure S6. ^{13}C NMR (CD_3OD, 400 MHz) of cyclic hexapeptide

Figure S7. The ^{13}C NMR (DEPT) spectrum of cyclic hexapeptide

Figure S8. The HMBC spectrum of cyclic hexapeptide

Figure S9. The ^{1}H-^{1}H COSY spectrum of cyclic hexapeptide

Figure S1. GC–MS Analysis of the BtD extract

Area Percent Report

Data File : C:\MSDCHEM\2\DATA\014-LGC.D Vial: 95
Acq On : 18 Oct 2022 9:59 Operator: M.G.M
Sample : BTZCDCl3 Splitless 40°C-1min, Inst : Instrumen
Misc : 10°C/min-250°C-5min,10°C/min-20min Multiplr: 1.00
Sample Amount: 1.00
MS Integration Params: autoint1.e

Signal : TIC

peak #	R.T. min	first scan	max scan	last scan	PK TY	peak height	corr. area	corr. % max.	% of total
1	6.893	117	124	137	M	220731	5563550	3.39%	1.208%
2	19.840	2087	2095	2121	BV	2252539	66055036	40.24%	14.343%
3	20.221	2143	2153	2165	VV 2	538038	12801791	7.80%	2.780%
4	20.924	2252	2260	2274	M	528670	13685068	8.34%	2.972%
5	21.193	2295	2301	2342	BB	974166	52105698	31.74%	11.314%
6	22.008	2417	2425	2434	VV	809337	15743630	9.59%	3.419%
7	25.055	2880	2889	2898	M	1138838	32704637	19.92%	7.102%
8	27.722	3288	3295	3310	VB	898734	30700493	18.70%	6.666%
9	28.826	3451	3463	3476	BB	1485641	42057954	25.62%	9.133%
10	32.780	4027	4065	4150	BB 3	848609	164147530	100.00%	35.643%
11	34.744	4350	4364	4384	VB 2	606289	24963514	15.21%	5.421%

Sum of corrected areas: 460528900

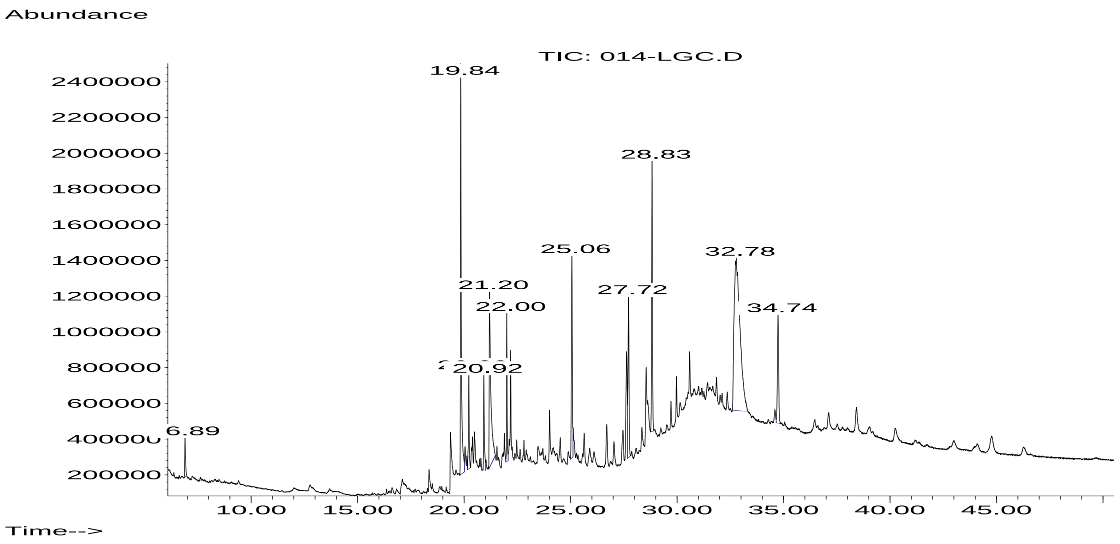

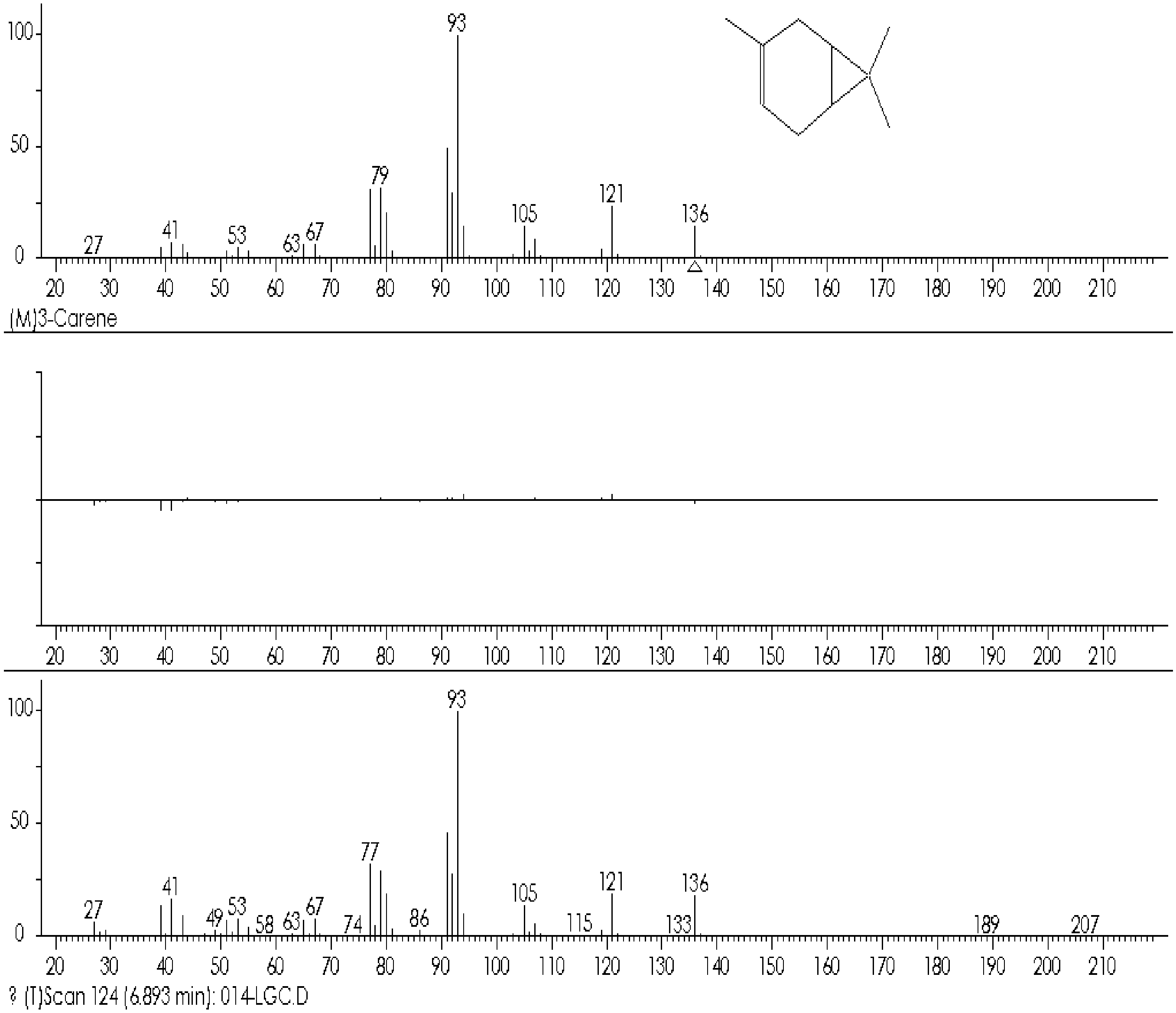

100
50
0
93
79
27
41
53
63
67
105
121
136
20 30 40 50 60 70 80 90 100 110 120 130 140 150 160 170 180 190 200 210
(M)3-Carene
20 30 40 50 60 70 80 90 100 110 120 130 140 150 160 170 180 190 200 210
100
50
0
93
77
27
41
49
53
58
63
67
74
86
105
115
121
133
136
189
207
20 30 40 50 60 70 80 90 100 110 120 130 140 150 160 170 180 190 200 210
? (T)Scan 124 (6.893 min): 014-LGC.D

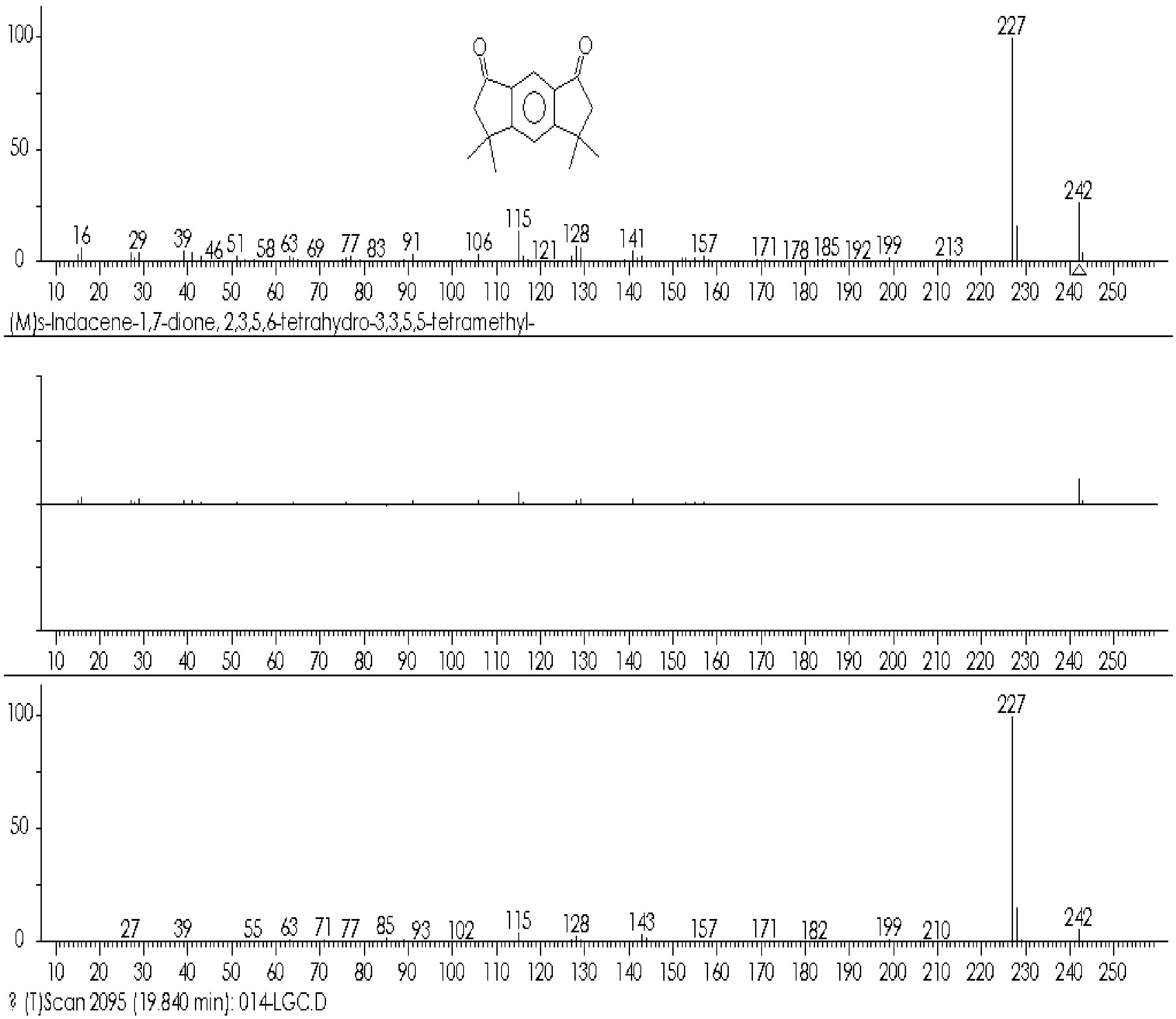

227
242
(M)s-Indacene-1,7-dione, 2,3,5,6-tetrahydro-3,3,5,5-tetramethyl-
227
242
? (T)Scan 2095 (19.840 min): 014-LGC.D

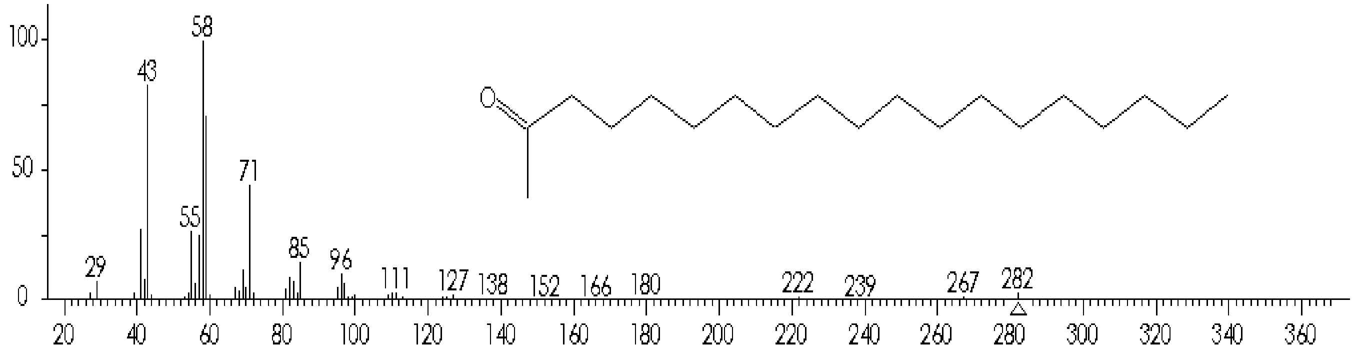

(M)2-Nonadecanone

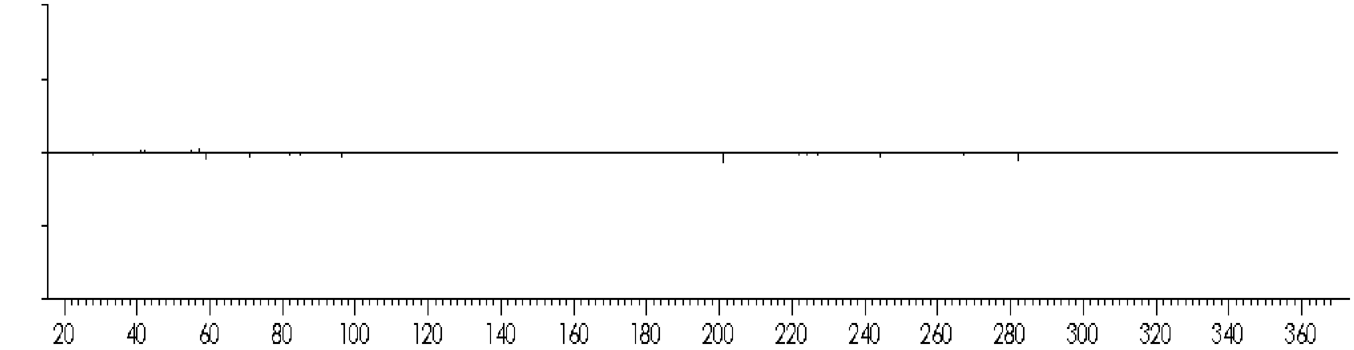

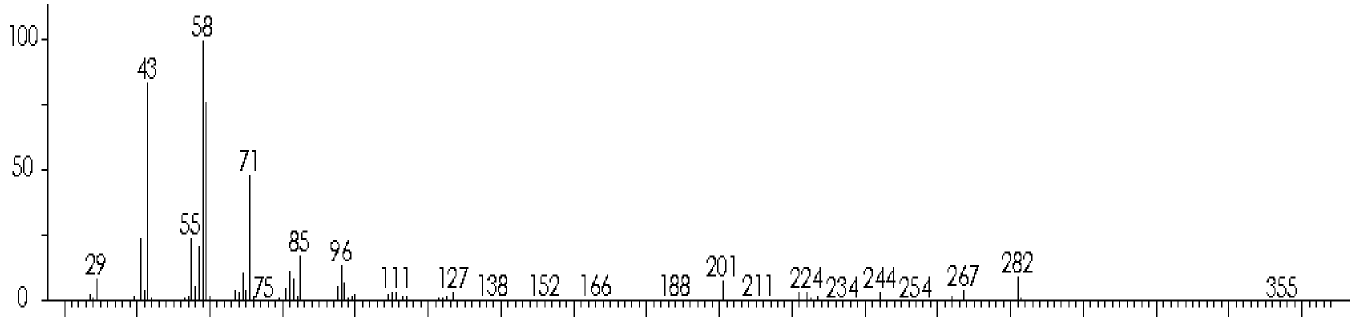

? (T)Scan 2153 (20.221 min): 014-LGC.D

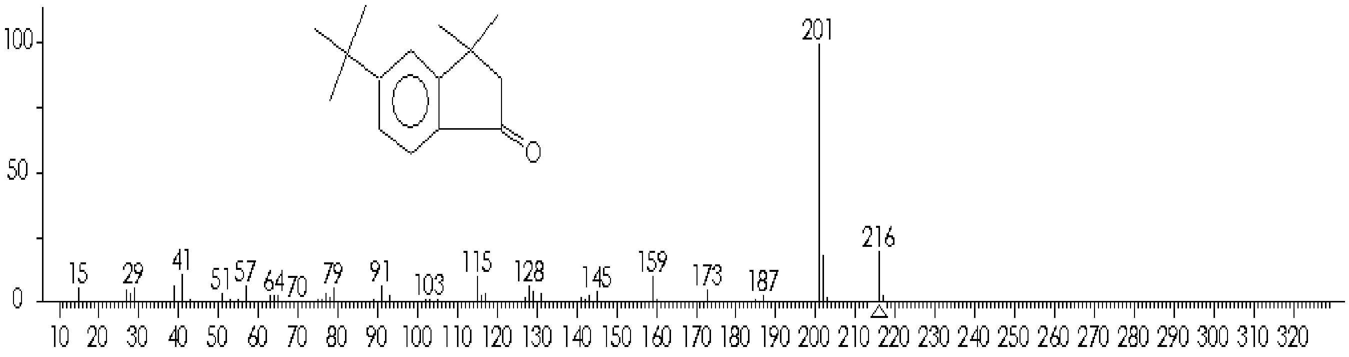

(M)1H-Inden-1-one, 5-(1,1-dimethylethyl)-2,3-dihydro-3,3-dimethyl-

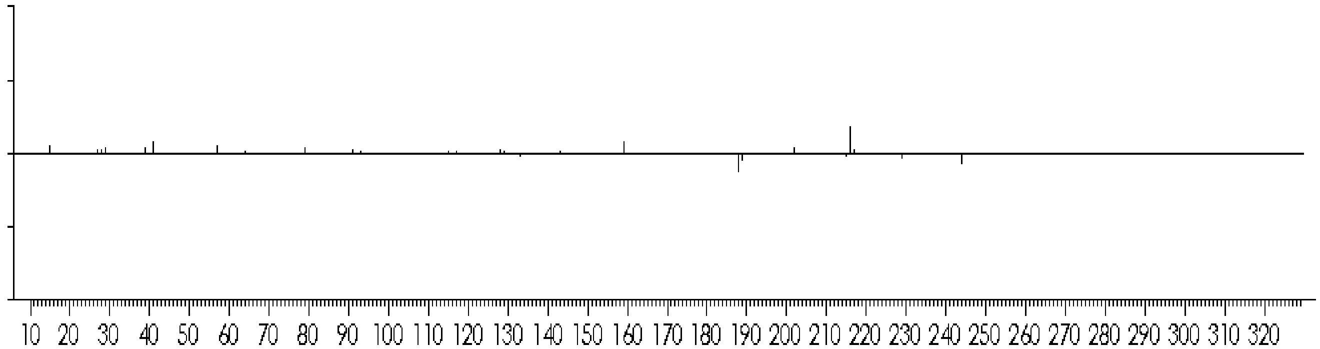

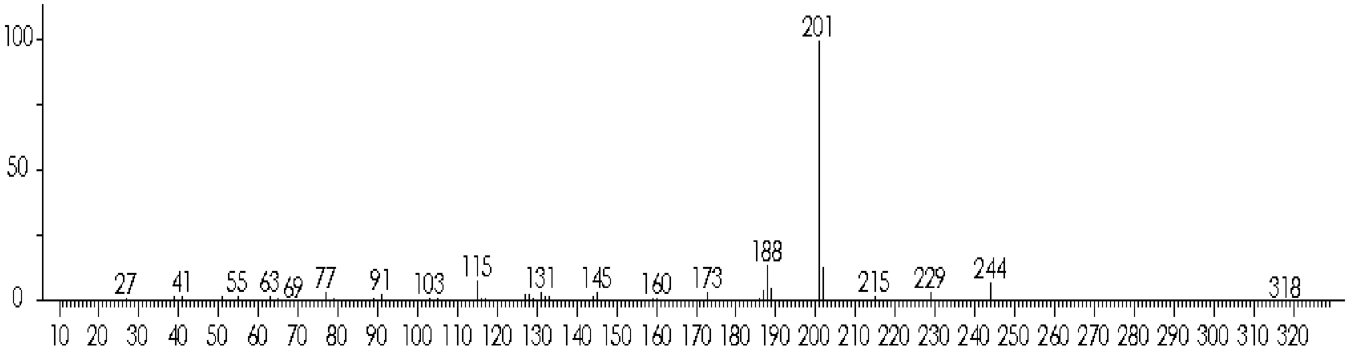

? (T)Scan 2302 (21.200 min): 014-LGC.D

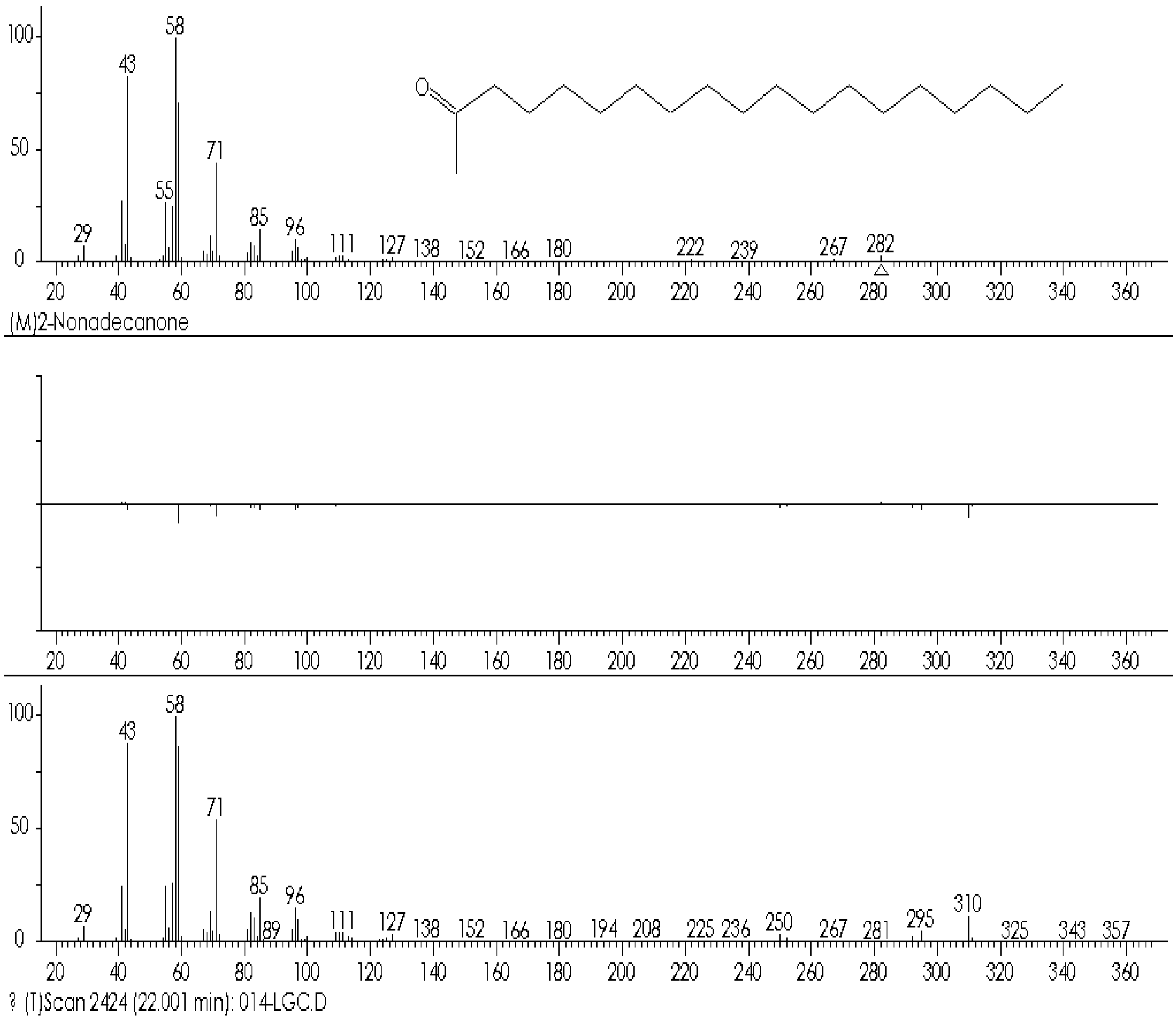

(M)2-Nonadecanone
? (T)Scan 2424 (22.001 min): 014-LGC.D

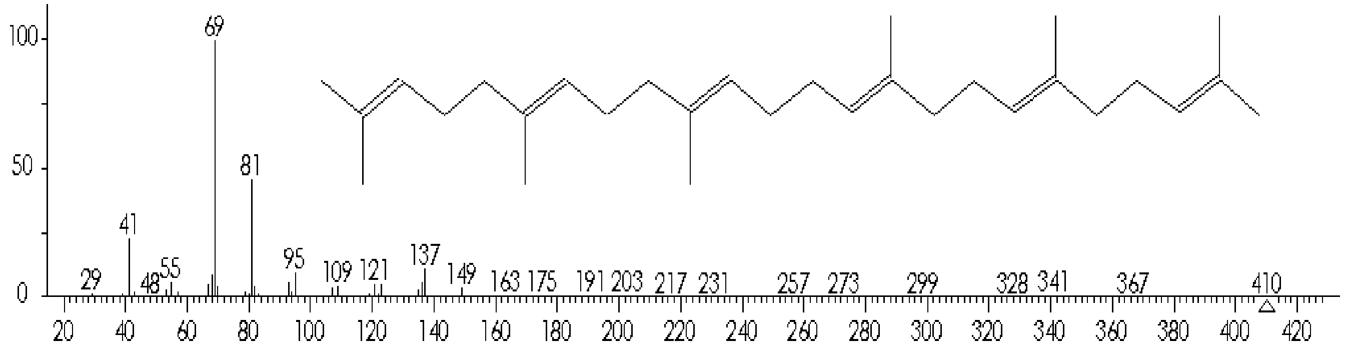

(R)Squalene

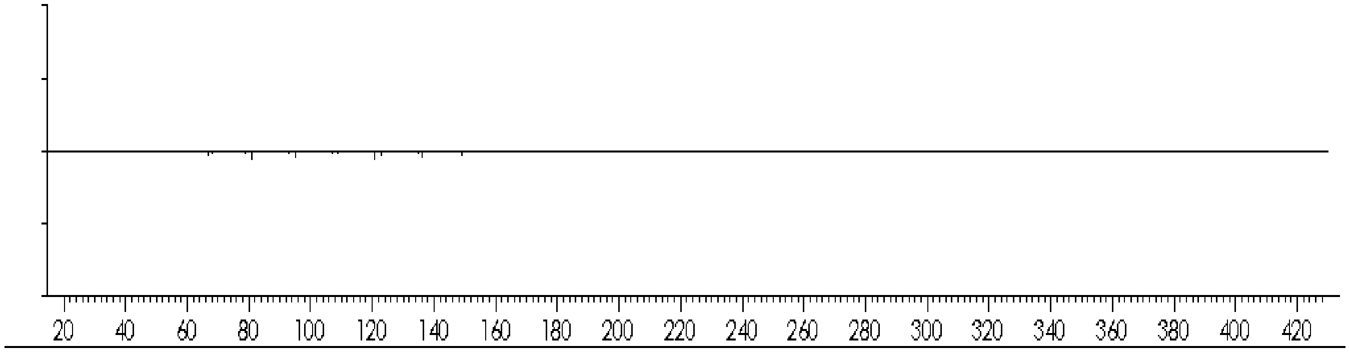

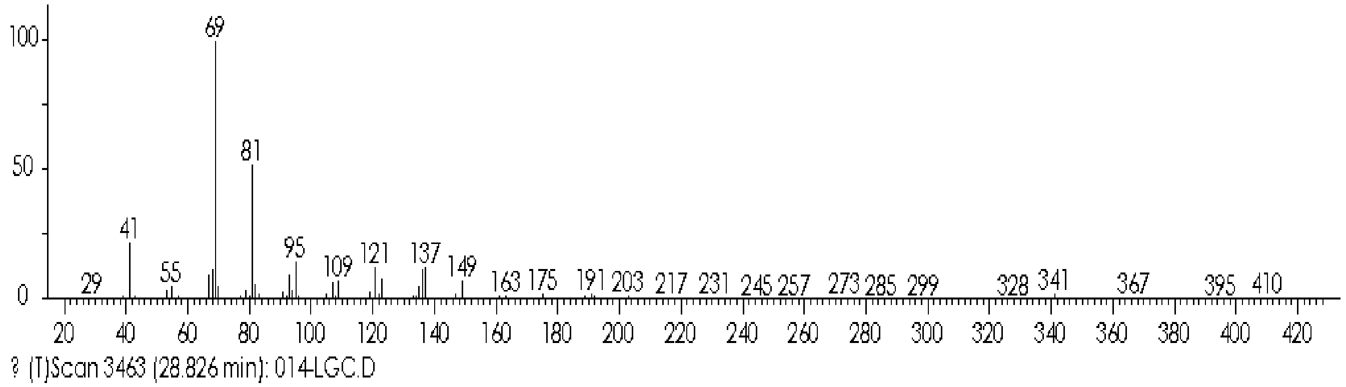

? (T)Scan 3463 (28.826 min): 014-LGC.D

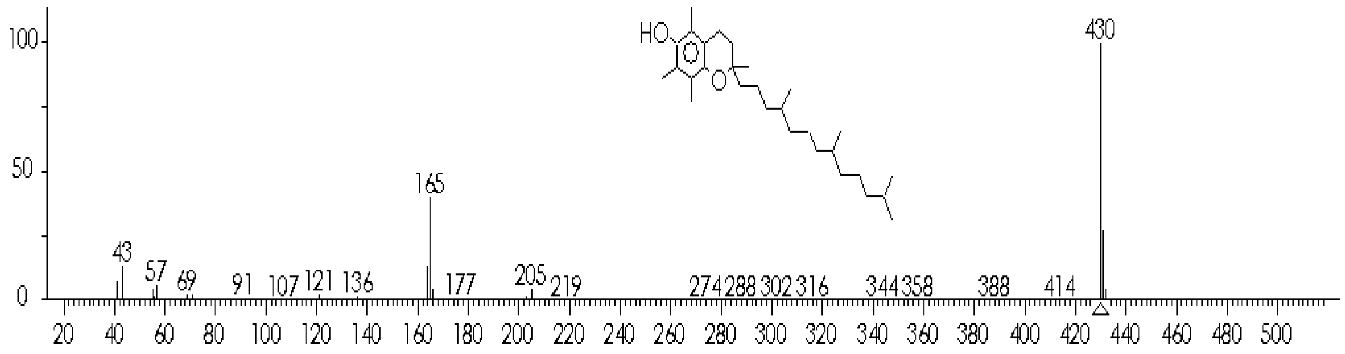

(M)D,α-Tocopherol

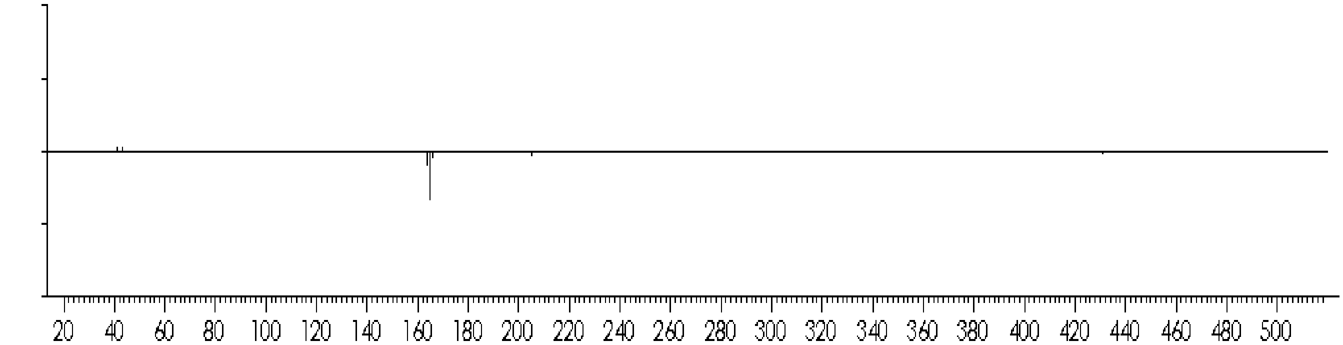

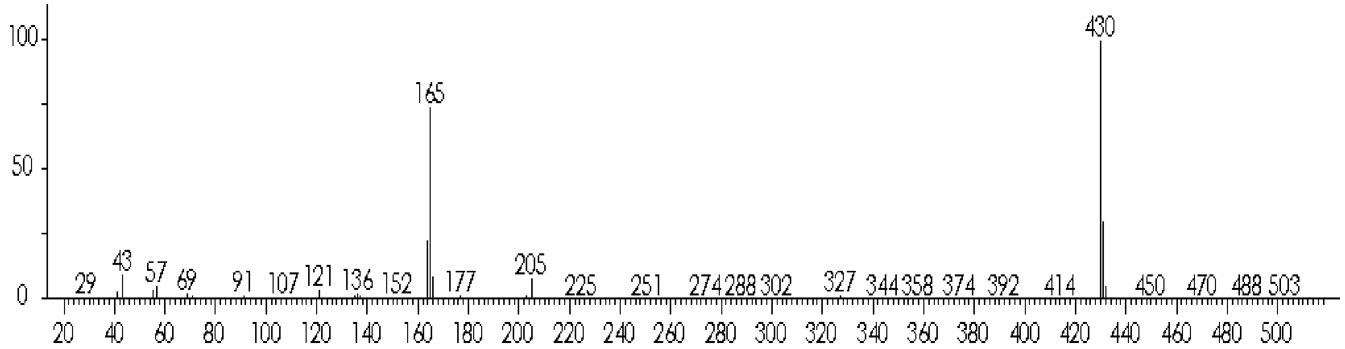

? (T)Scan 4065 (32.780 min): 014-LGC.D

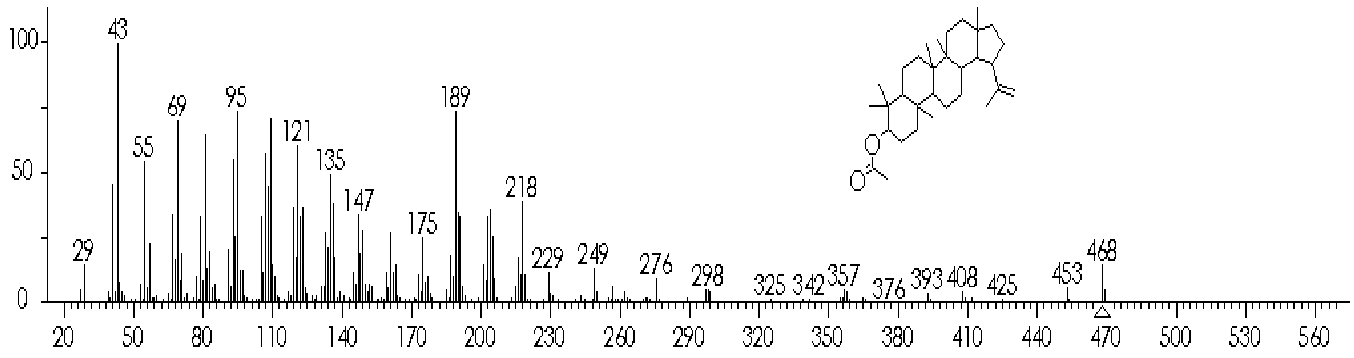

(M)Lup-20(29)-en-3-ol, acetate, (3β)-

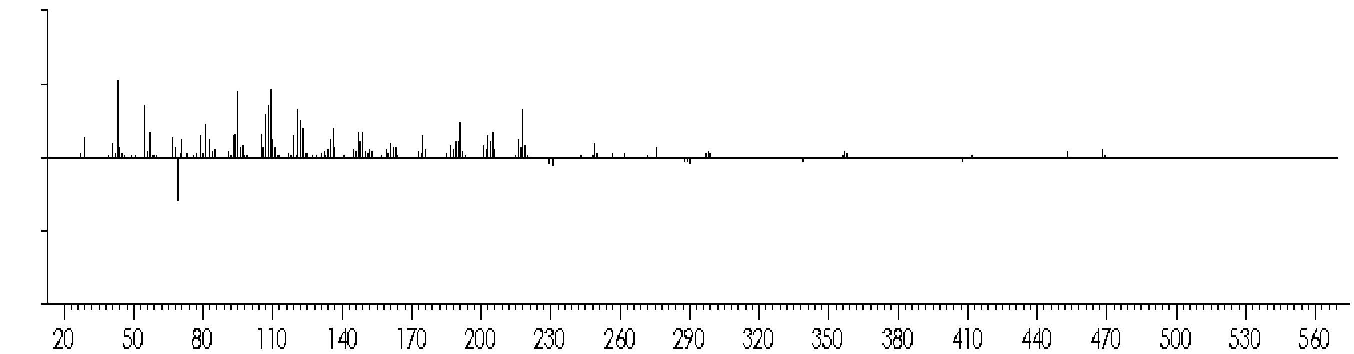

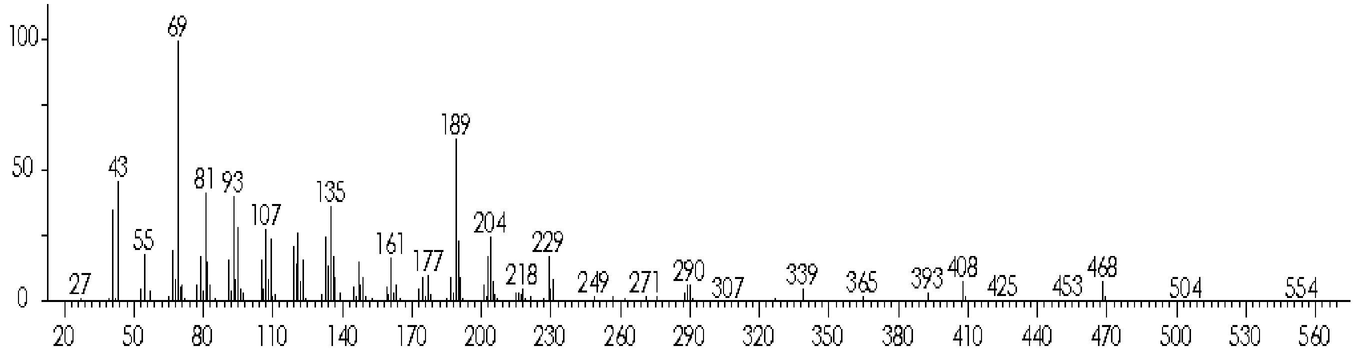

? (T)Scan 4363 (34.738 min): 014-LGC.D

Figure S2. GC–MS Analysis of the BtH extract

Area Percent Report

Data File : C:\MSDCHEM\2\DATA\015-LGC.D Vial: 96
Acq On : 18 Oct 2022 11:15 Operator: M.G.M
Sample : BtzHex Splitless 40°C-1min, Inst : Instrumen
Misc : 10°C/min-250°C-5min,10°C/min-20min Multiplr: 1.00
Sample Amount: 1.00
MS Integration Params: autoint1.e

Signal : TIC

peak #	R.T. min	first scan	max scan	last scan	PK TY	peak height	corr. area	corr. % max.	% of total
1	18.493	1870	1890	1910	BB	1054859	23320727	7.17%	3.216%
2	19.150	1986	1990	1994	M	371667	5936264	1.83%	0.819%
3	20.122	2133	2138	2142	M	1521626	25098555	7.72%	3.462%
4	20.175	2143	2146	2150	M3	988264	18038391	5.55%	2.488%
5	20.398	2176	2180	2185	M	284165	4668471	1.44%	0.644%
6	20.727	2224	2230	2234	M	513857	8499903	2.61%	1.172%
7	20.773	2235	2237	2245	M2	478580	10314151	3.17%	1.422%
8	22.474	2473	2496	2508	BB	1397806	27913041	8.58%	3.850%
9	22.907	2558	2562	2568	M2	244262	4606328	1.42%	0.635%
10	26.704	3120	3140	3164	BB 3	500167	24591938	7.56%	3.392%
11	28.845	3446	3466	3496	BB	4676899	145442645	44.72%	20.059%
12	29.719	3580	3599	3616	BB	811542	20855076	6.41%	2.876%
13	32.681	4025	4050	4122	BB	6757043	325236826	100.00%	44.855%
14	35.657	4479	4503	4536	BB 4	575766	35057274	10.78%	4.835%
15	44.774	5878	5891	5920	M4	480756	45497576	13.99%	6.275%

Sum of corrected areas: 725077165

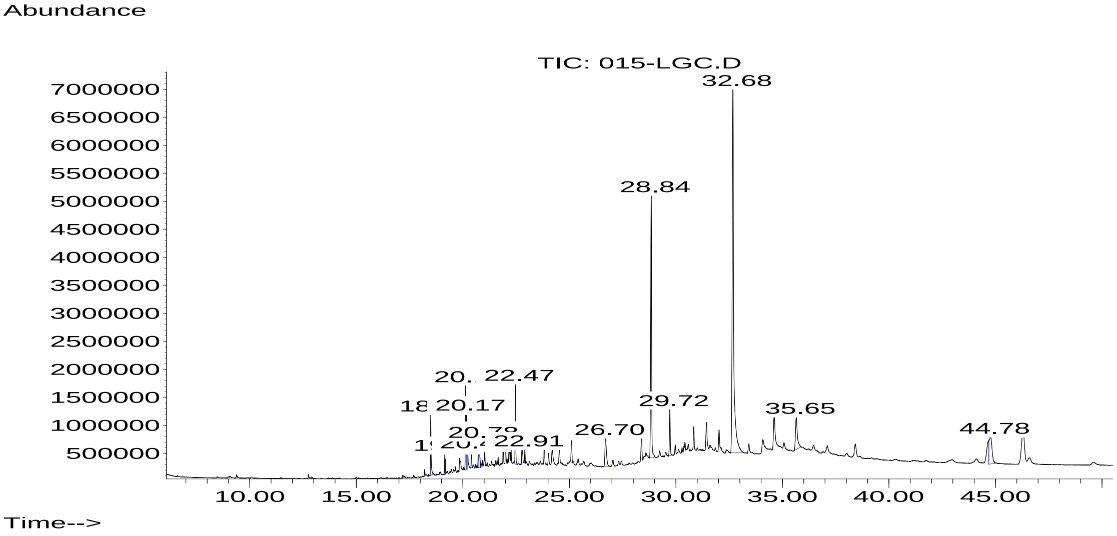

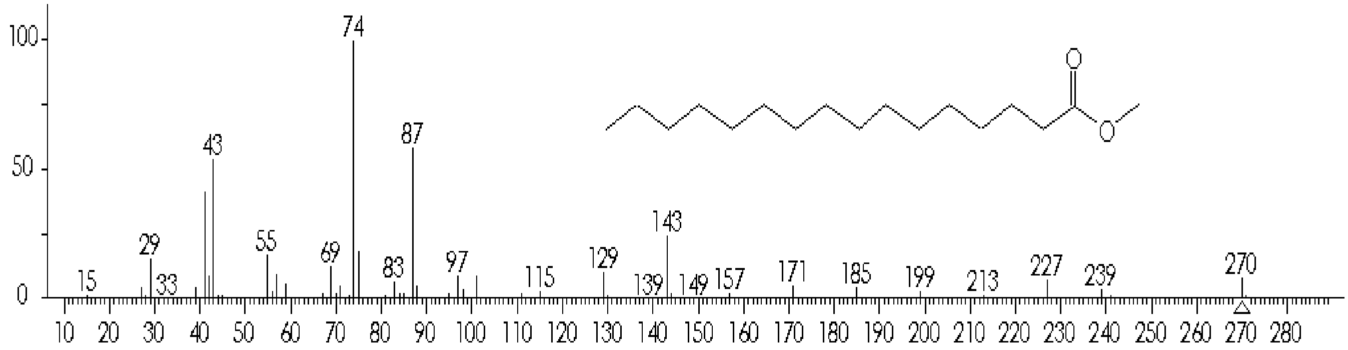

(R)Hexadecanoic acid, methyl ester

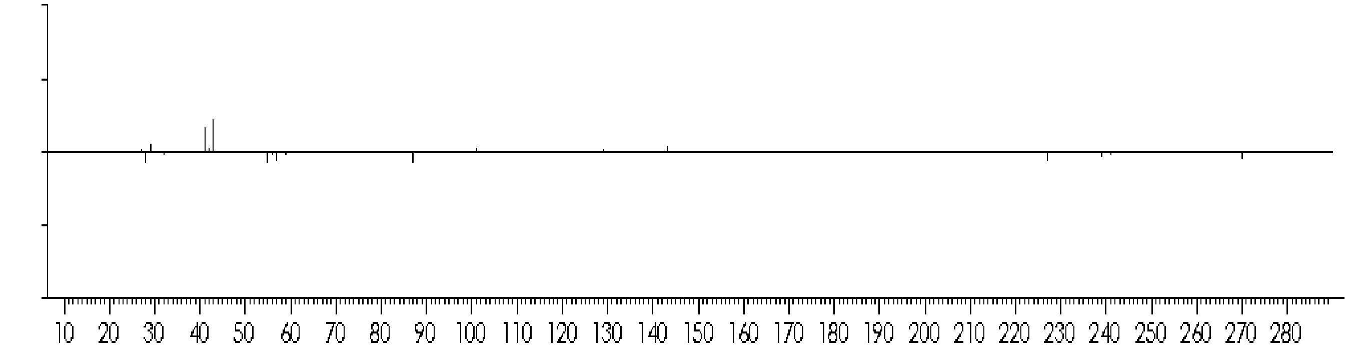

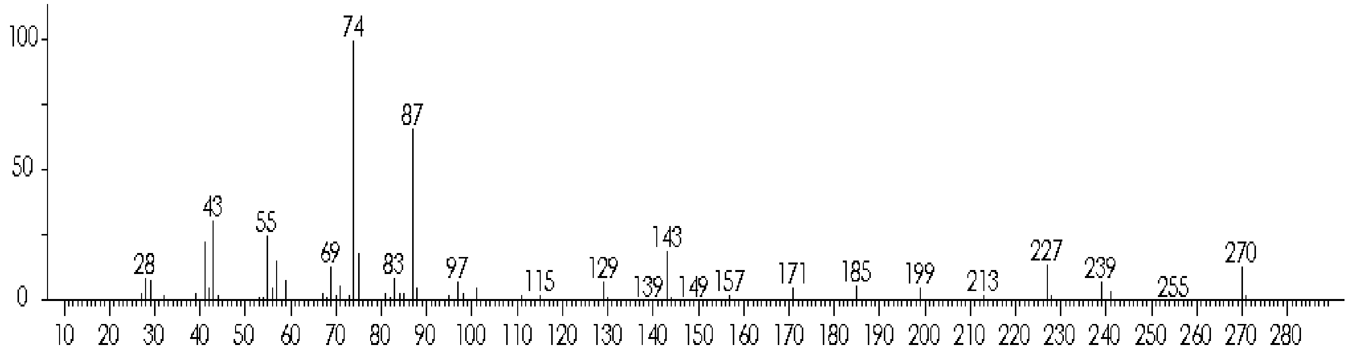

? (T)Scan 1890 (18.493 min): 015-LGC.D

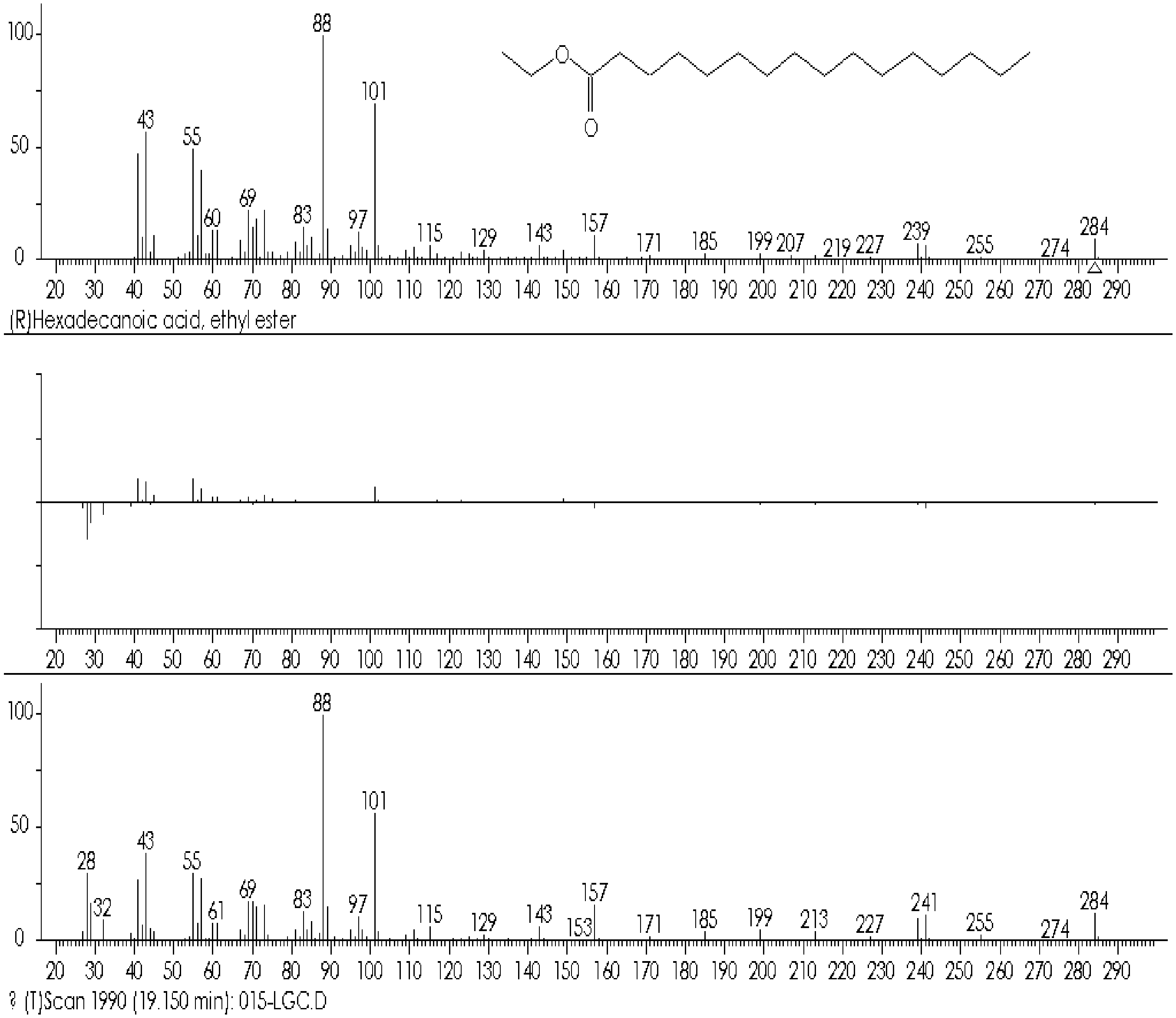

100
50
0
88
101
43
55
60
69
83
97
115
129
143
157
171
185
199
207
219
227
239
255
274
284
O
O
20 30 40 50 60 70 80 90 100 110 120 130 140 150 160 170 180 190 200 210 220 230 240 250 260 270 280 290
(R)Hexadecanoic acid, ethyl ester
20 30 40 50 60 70 80 90 100 110 120 130 140 150 160 170 180 190 200 210 220 230 240 250 260 270 280 290
100
50
0
88
101
28
32
43
55
61
69
83
97
115
129
143
153
157
171
185
199
213
227
241
255
274
284
20 30 40 50 60 70 80 90 100 110 120 130 140 150 160 170 180 190 200 210 220 230 240 250 260 270 280 290
? (T)Scan 1990 (19.150 min): 015-LGC.D

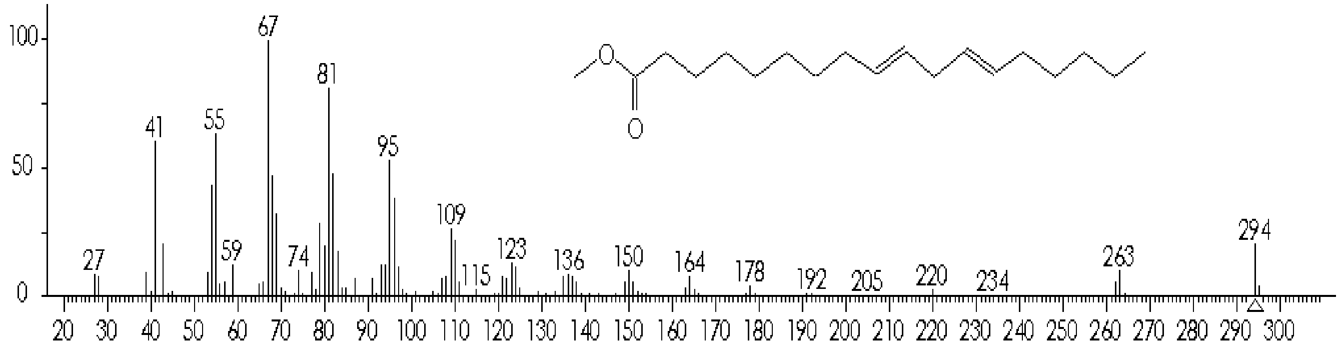

(M)9,12-Octadecadienoic acid (Z,Z)-, methyl ester

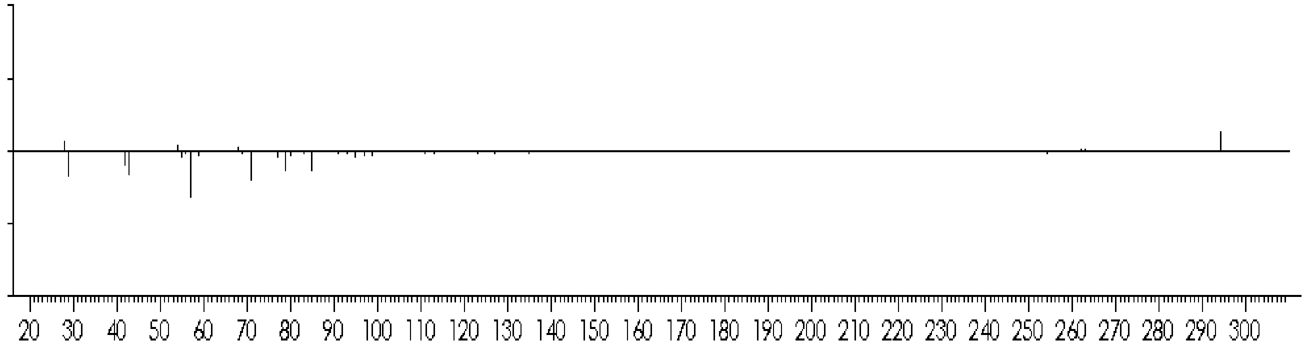

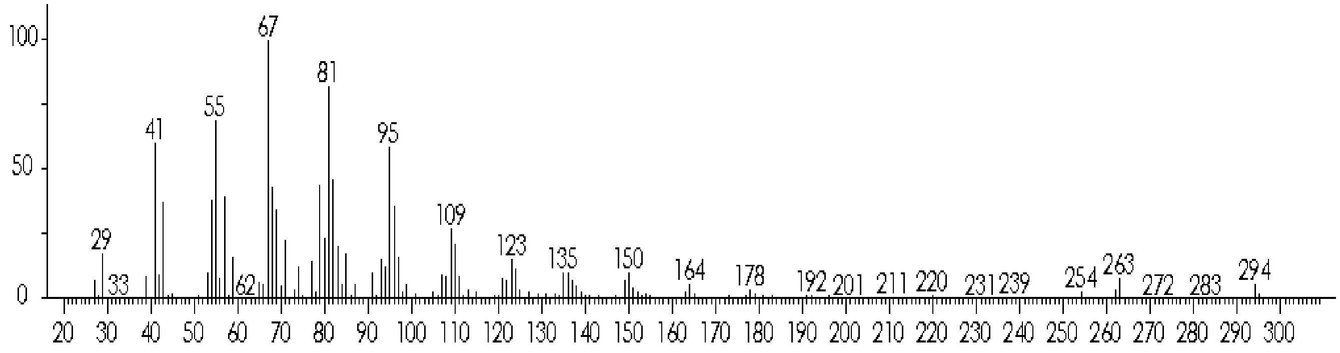

? (T)Scan 2138 (20.122 min): 015-LGC.D

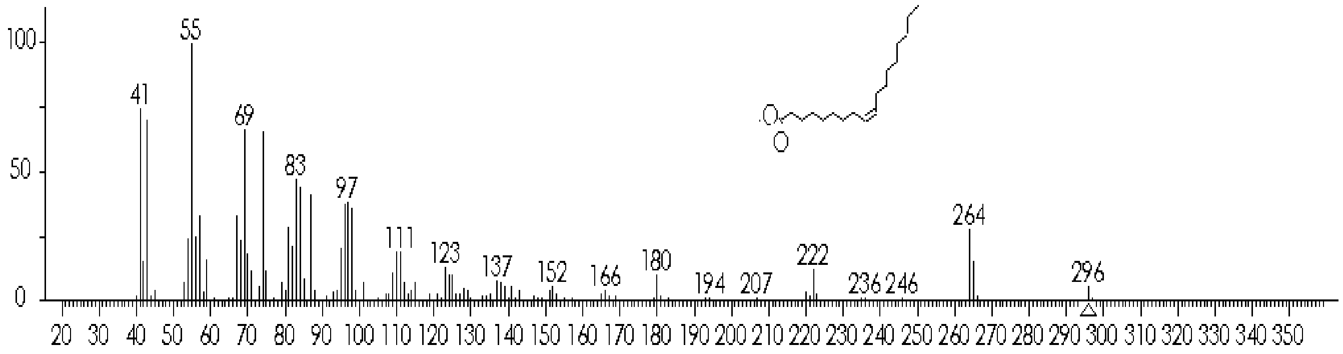

(R)9-Octadecenoic acid (Z)-, methyl ester

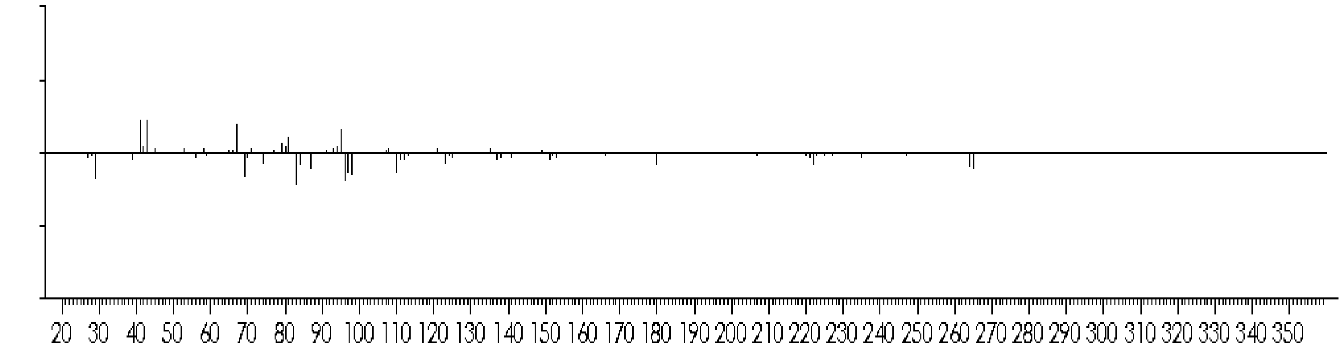

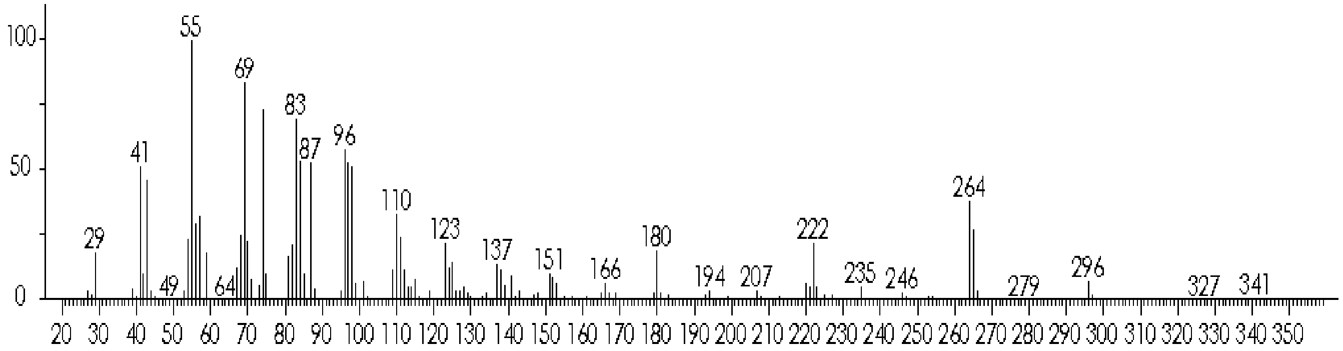

? (T)Scan 2146 (20.175 min): 015-LGC.D

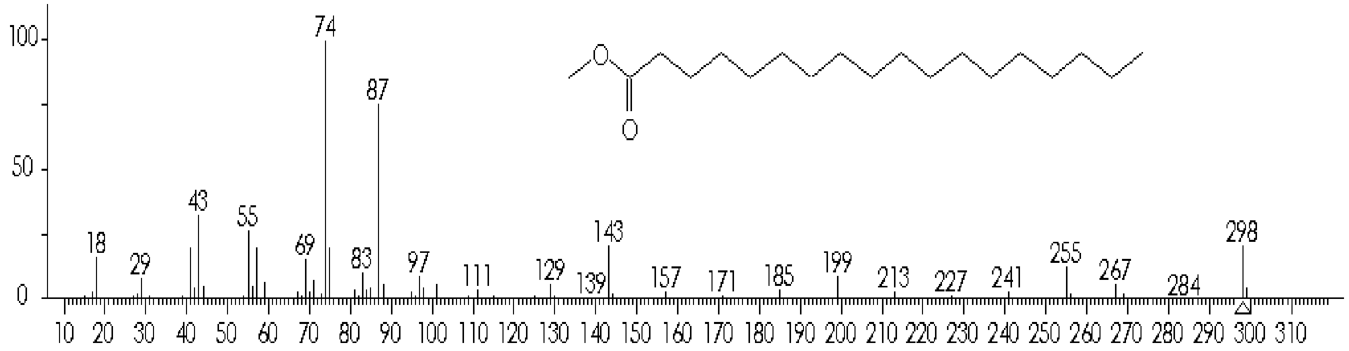

(R)Octadecanoic acid, methyl ester

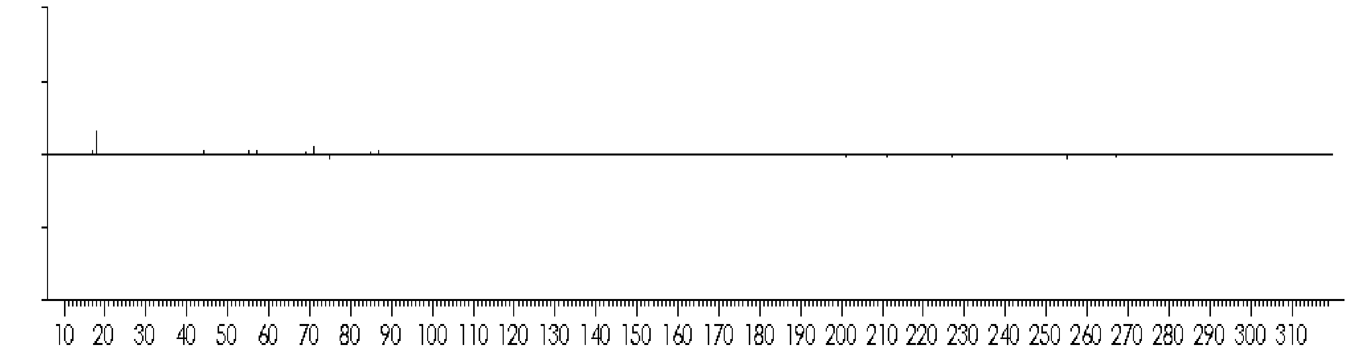

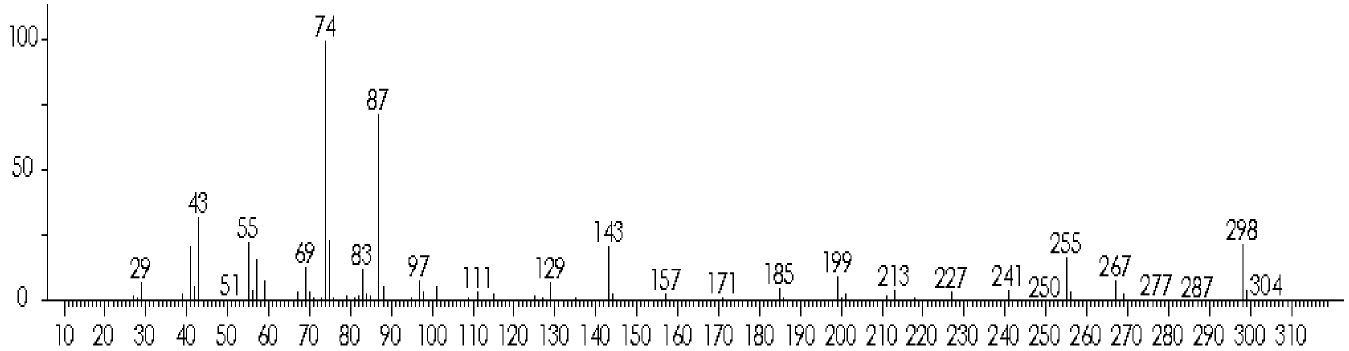

? (T)Scan 2181 (20.405 min): 015-LGC.D

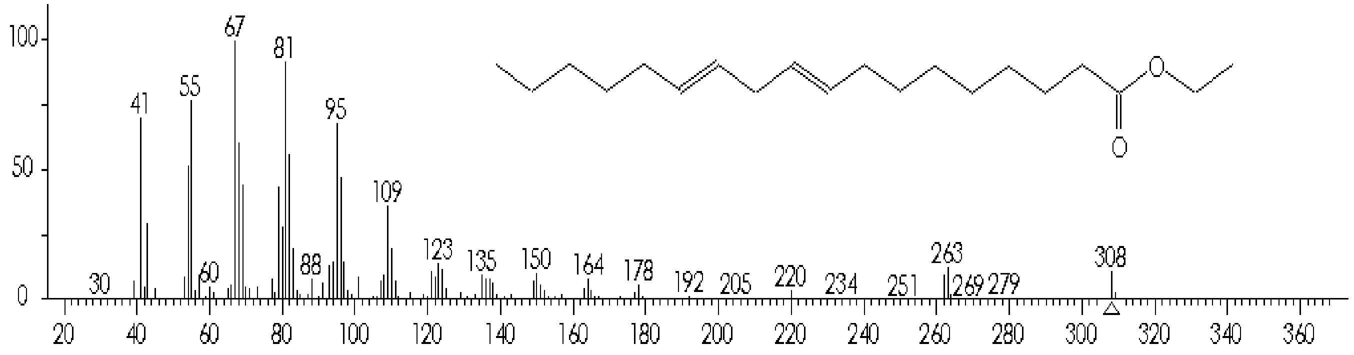

(M)9,12-Octadecadienoic acid, ethyl ester

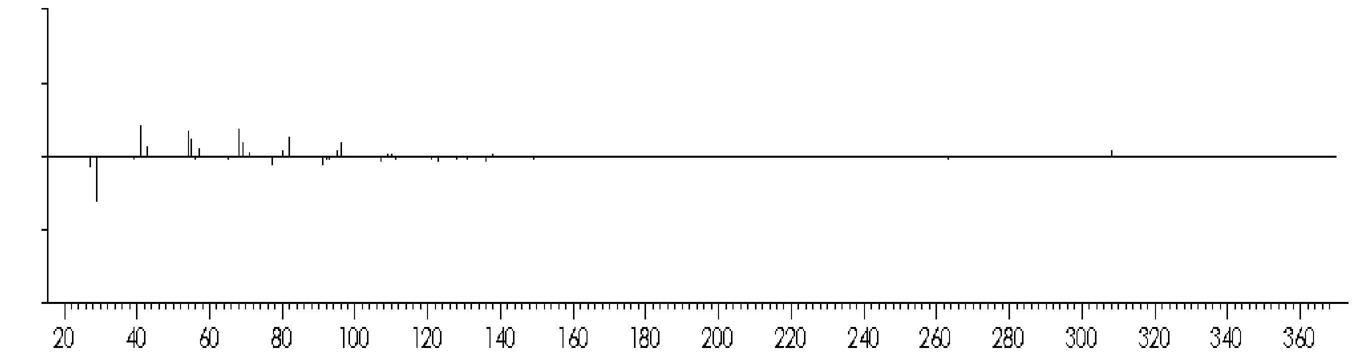

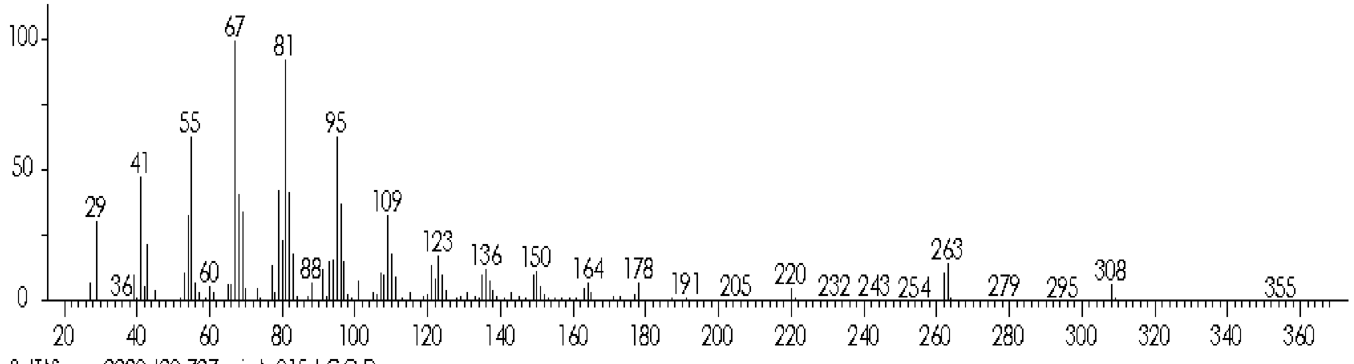

? (T)Scan 2230 (20.727 min): 015-LGC.D

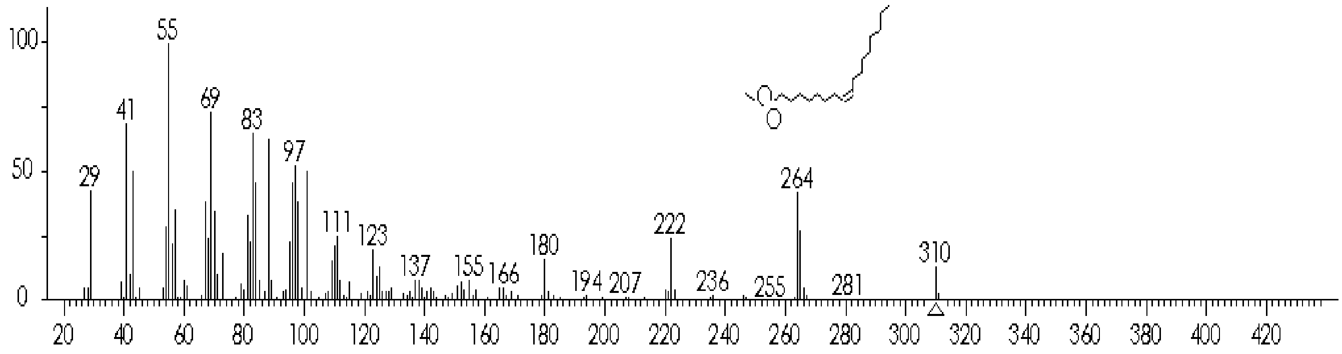

(R)Ethyl Oleate

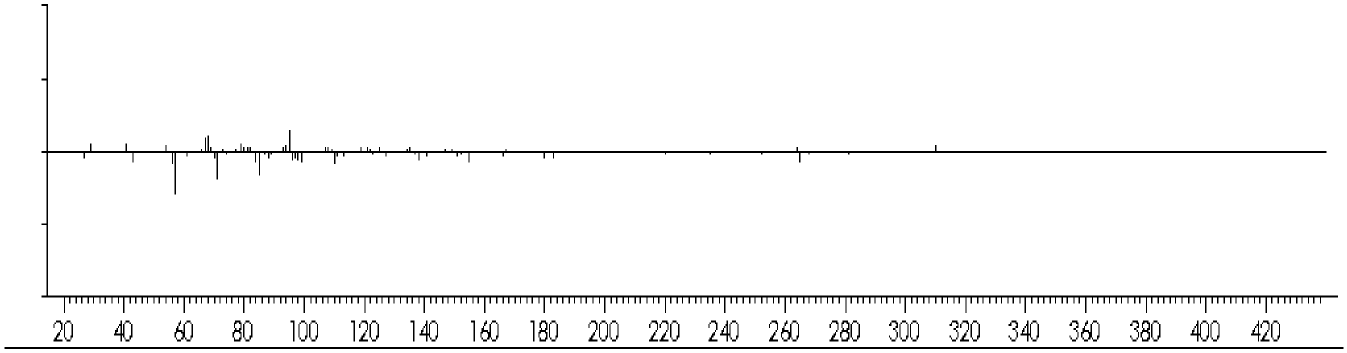

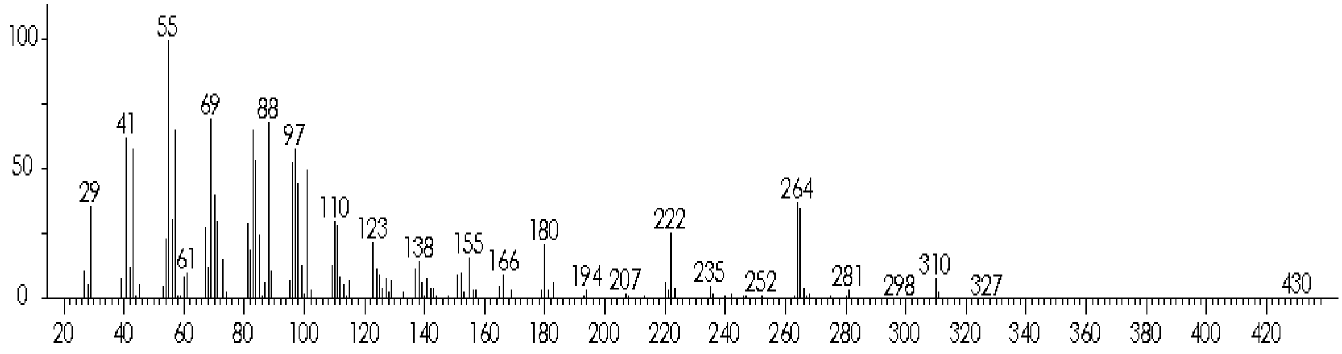

? (T)Scan 2237 (20.773 min): 015-LGC.D

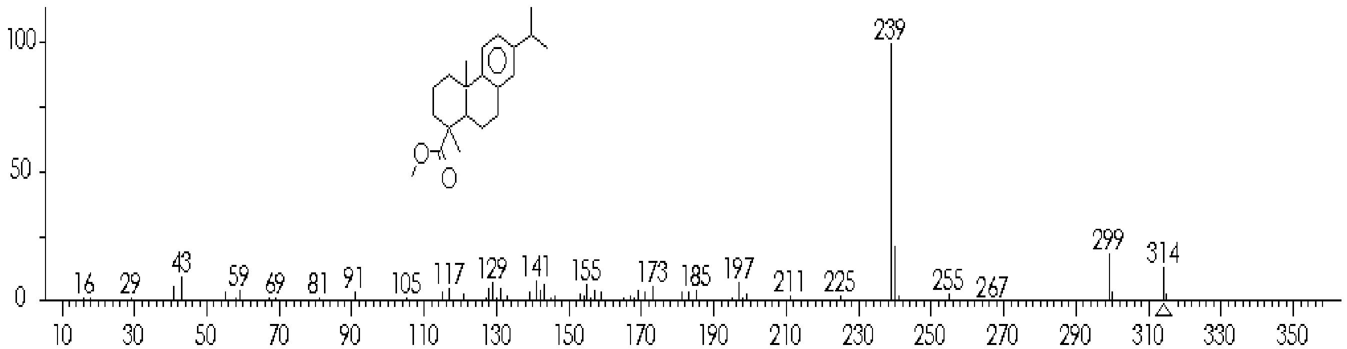

(M)1-Phenanthrenecarboxylic acid, 1,2,3,4,4a,9,10,10a-octahydro-1,4a-dimethyl-7-(1-methylethyl)-, methyl ester, [1R-(1α,4aβ,10aα)]-

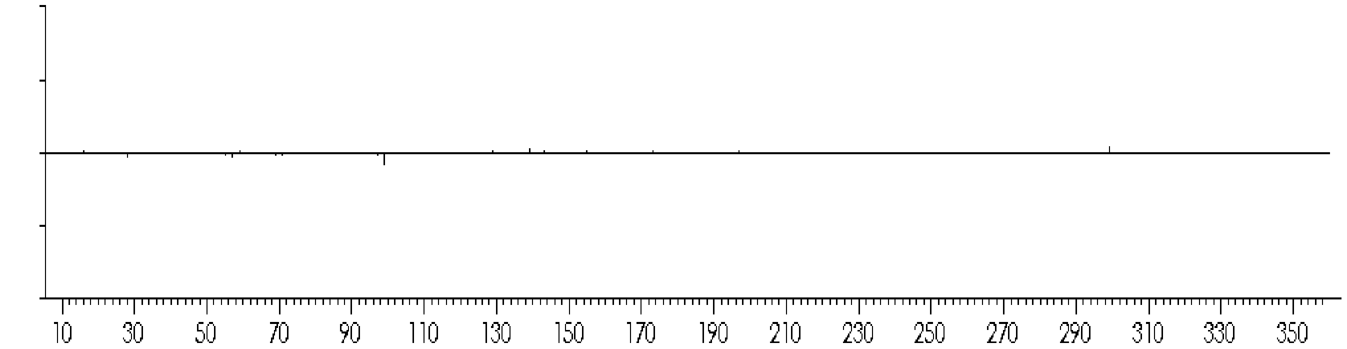

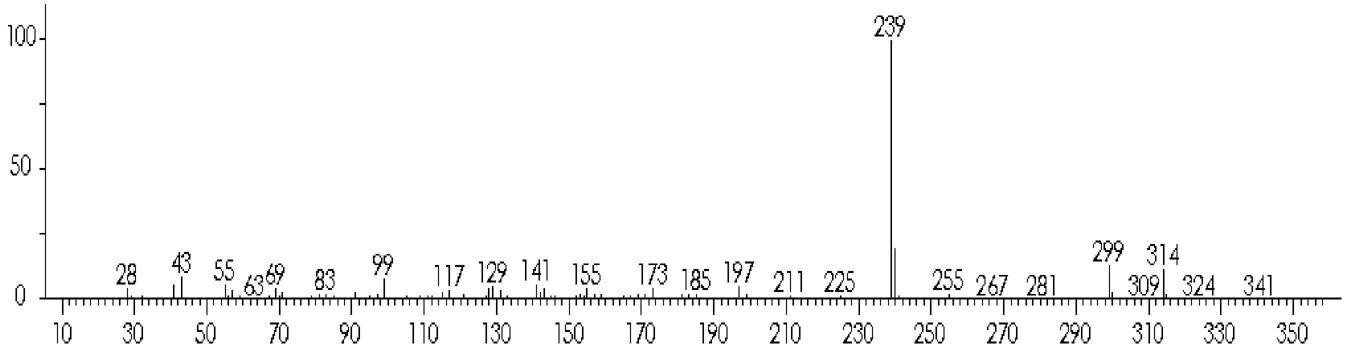

? (T)Scan 2495 (22.467 min): 015-LGC.D

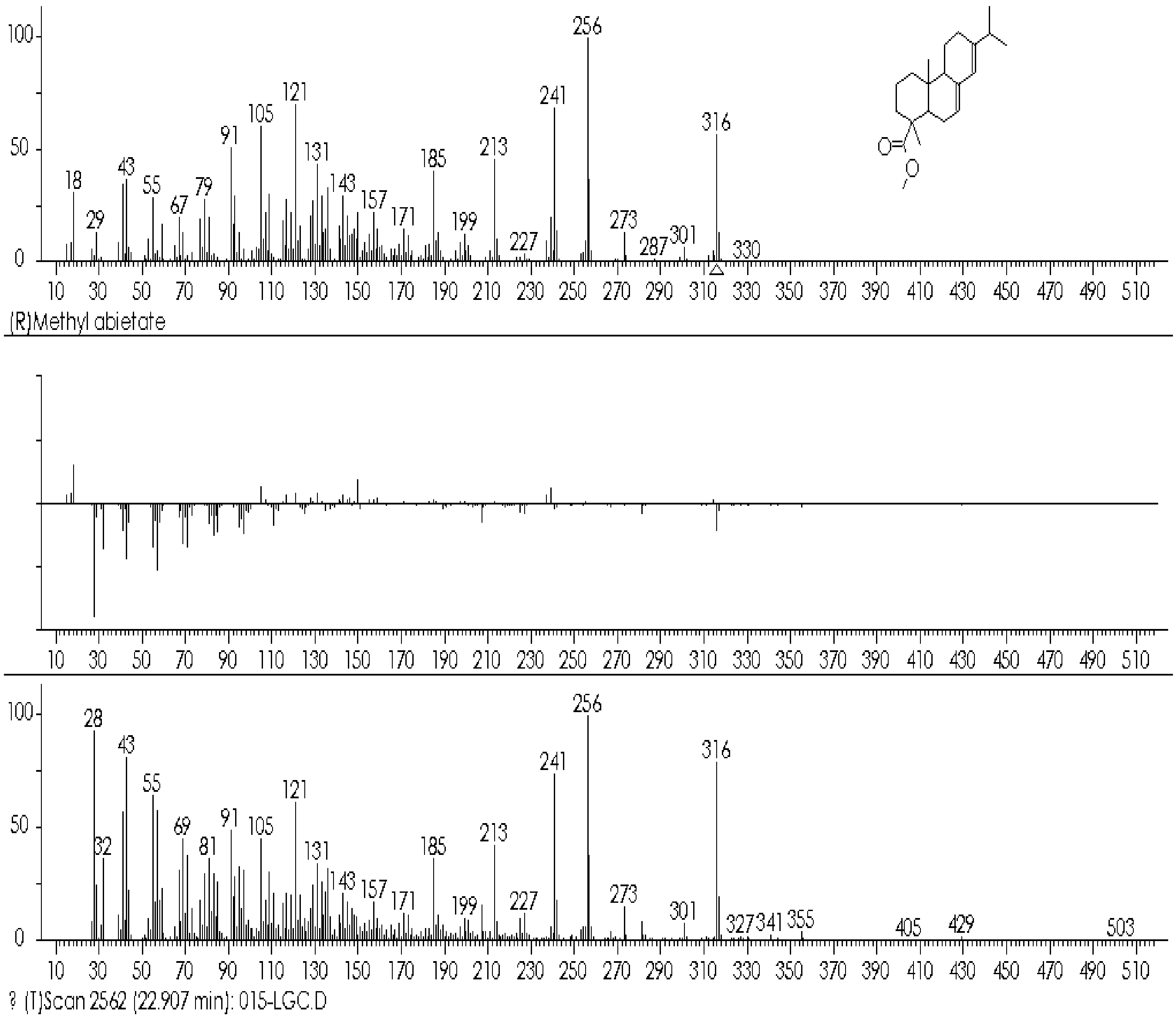
(R)Methyl abietate
? (T)Scan 2562 (22.907 min): 015-LGC.D

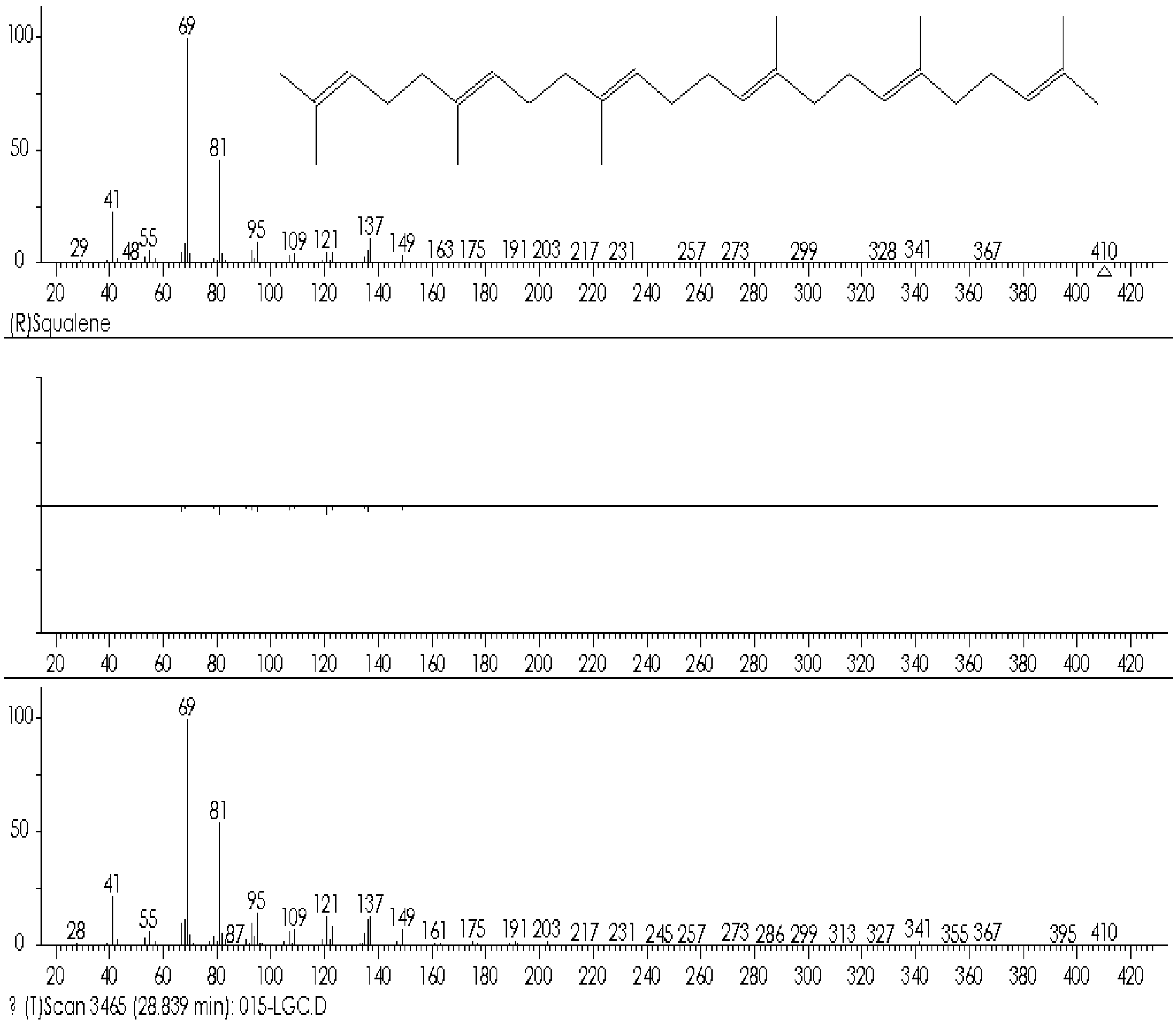

100
50
0
69
81
41
29
48
55
95
109
121
137
149
163
175
191
203
217
231
257
273
299
328
341
367
410
20 40 60 80 100 120 140 160 180 200 220 240 260 280 300 320 340 360 380 400 420
(R)Squalene
20 40 60 80 100 120 140 160 180 200 220 240 260 280 300 320 340 360 380 400 420
100
50
0
69
81
41
28
55
87
95
109
121
137
149
161
175
191
203
217
231
245
257
273
286
299
313
327
341
355
367
395
410
20 40 60 80 100 120 140 160 180 200 220 240 260 280 300 320 340 360 380 400 420
? (T)Scan 3465 (28.839 min): 015-LGC.D

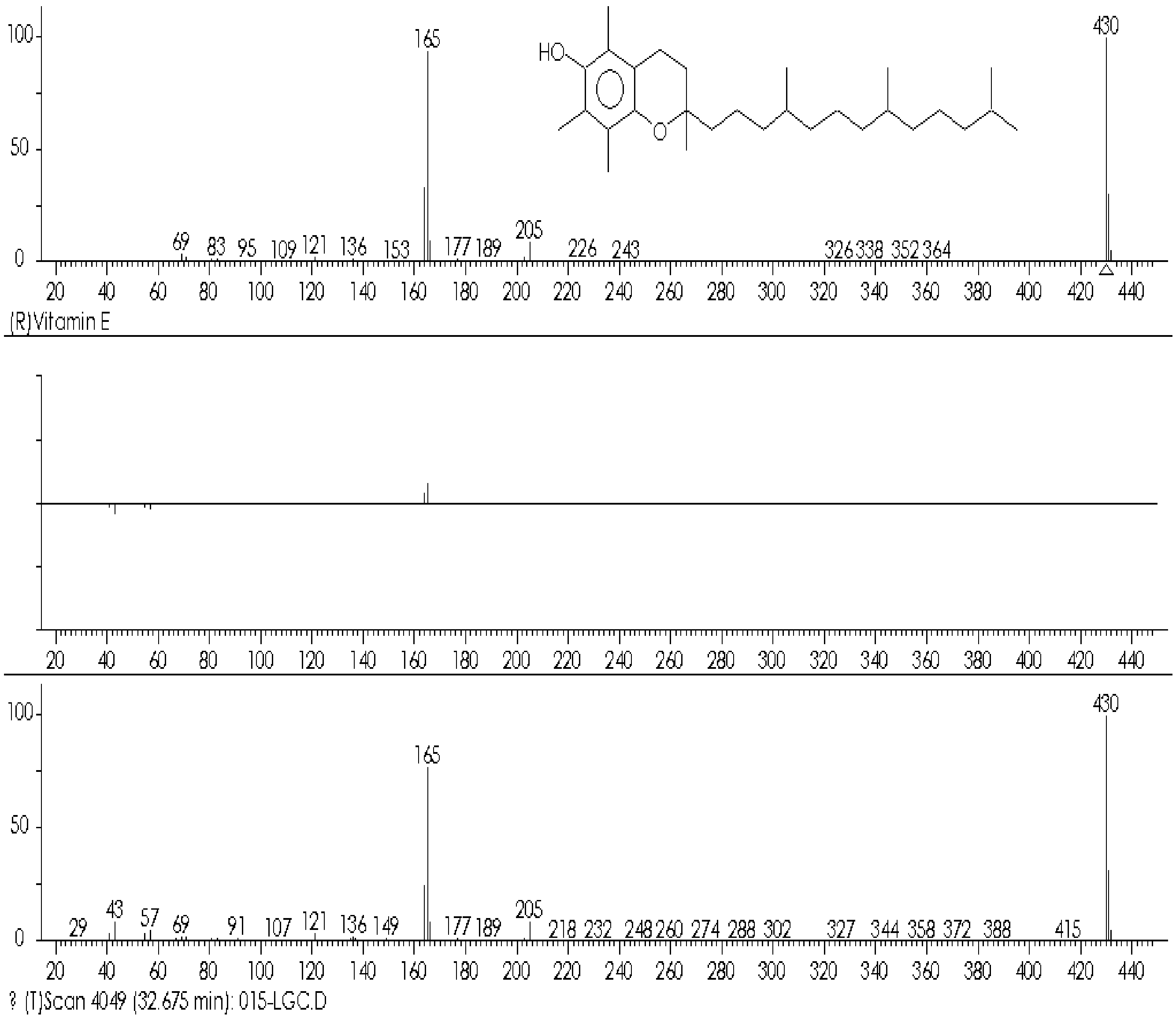
HO
O
100
50
0
69 83 95 109 121 136 153 165 177 189 205 226 243 326 338 352 364 430
20 40 60 80 100 120 140 160 180 200 220 240 260 280 300 320 340 360 380 400 420 440
(R)Vitamin E
20 40 60 80 100 120 140 160 180 200 220 240 260 280 300 320 340 360 380 400 420 440
100
50
0
29 43 57 69 91 107 121 136 149 165 177 189 205 218 232 248 260 274 288 302 327 344 358 372 388 415 430
20 40 60 80 100 120 140 160 180 200 220 240 260 280 300 320 340 360 380 400 420 440
? (T)Scan 4049 (32.675 min): 015-LGC.D

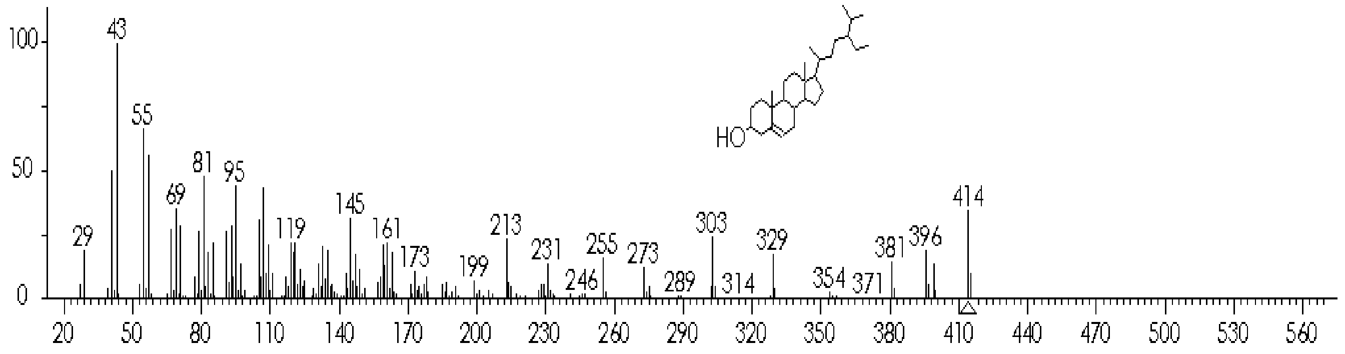

(R)τ-Sitosterol

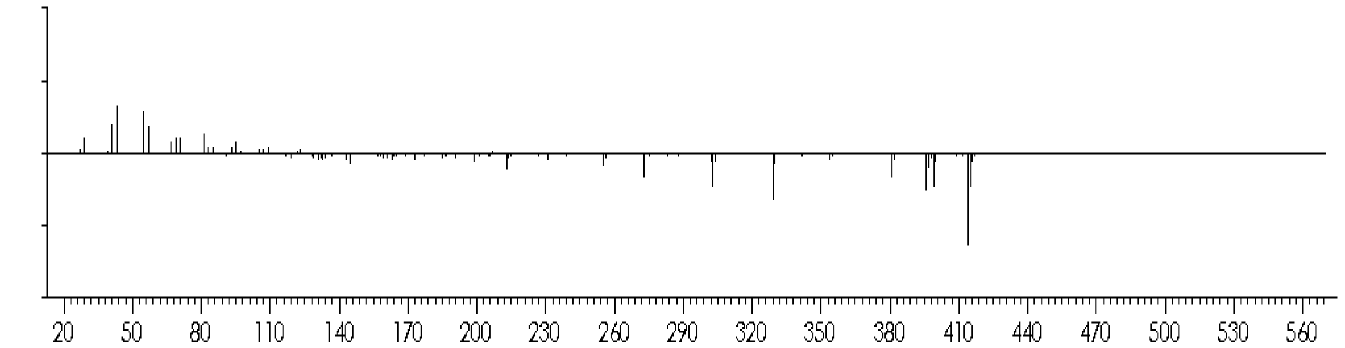

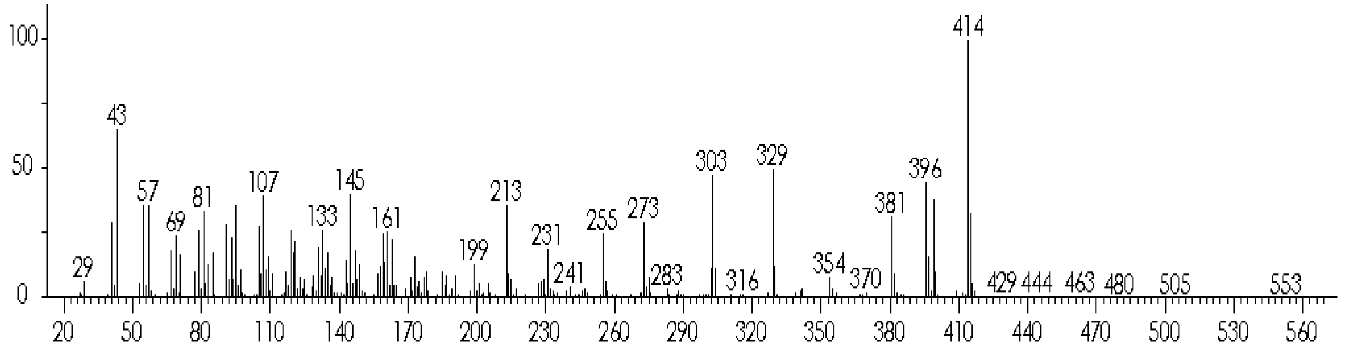

? (T)Scan 4503 (35.657 min): 015-LGC.D

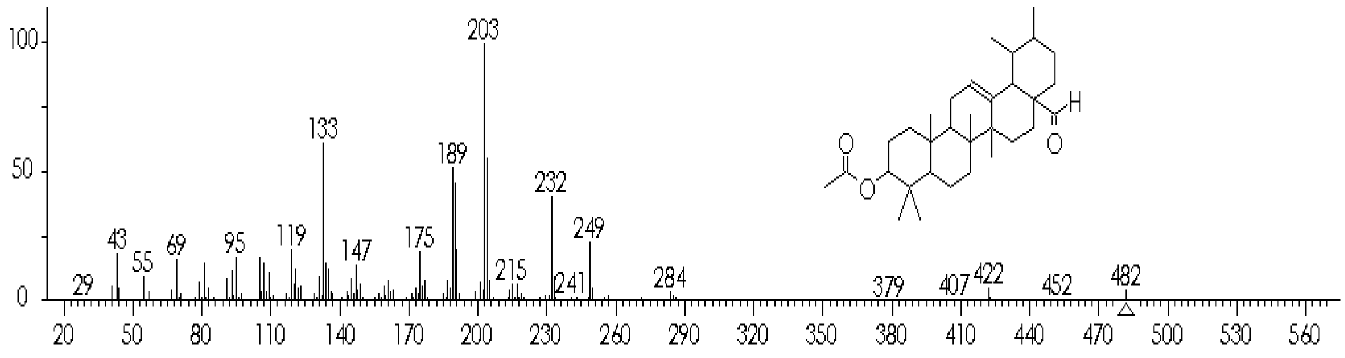

(M)Urs-12-en-28-al, 3-(acetyloxy)-, (3β)-

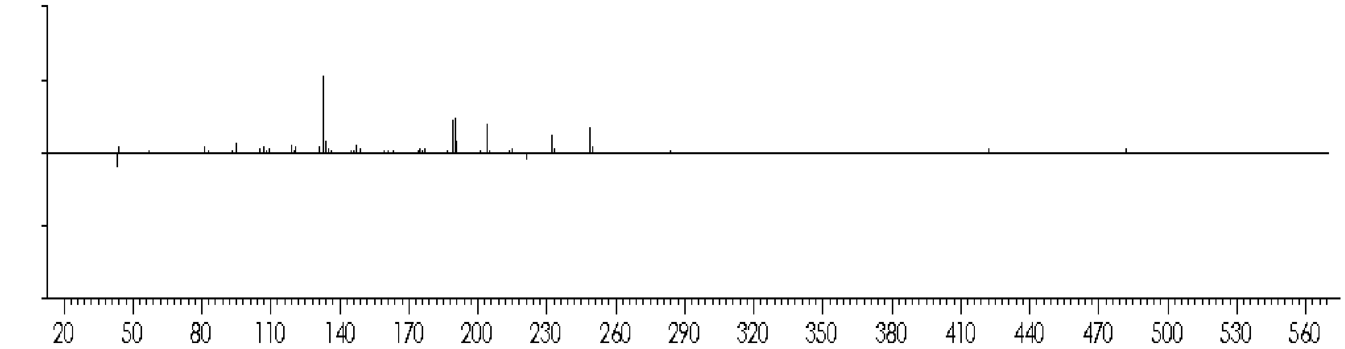

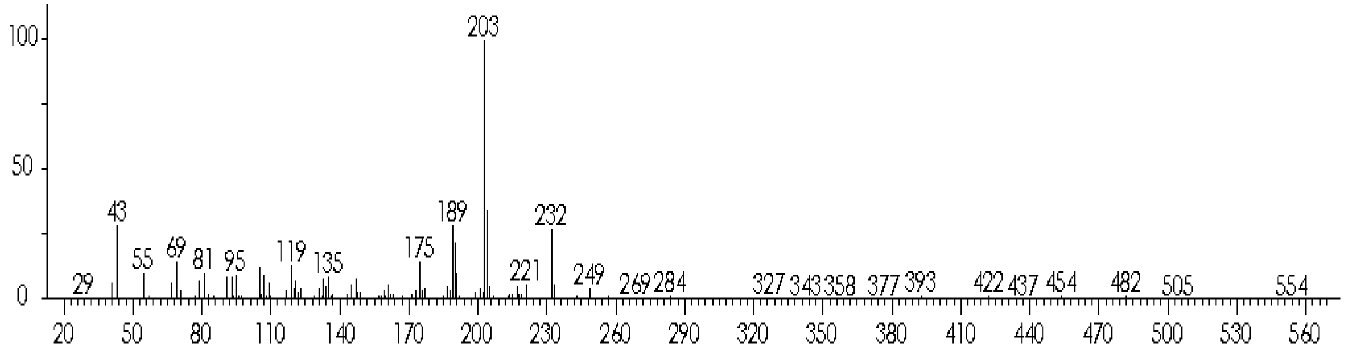

? (T)Scan 5892 (44.781 min): 015-LGC.D

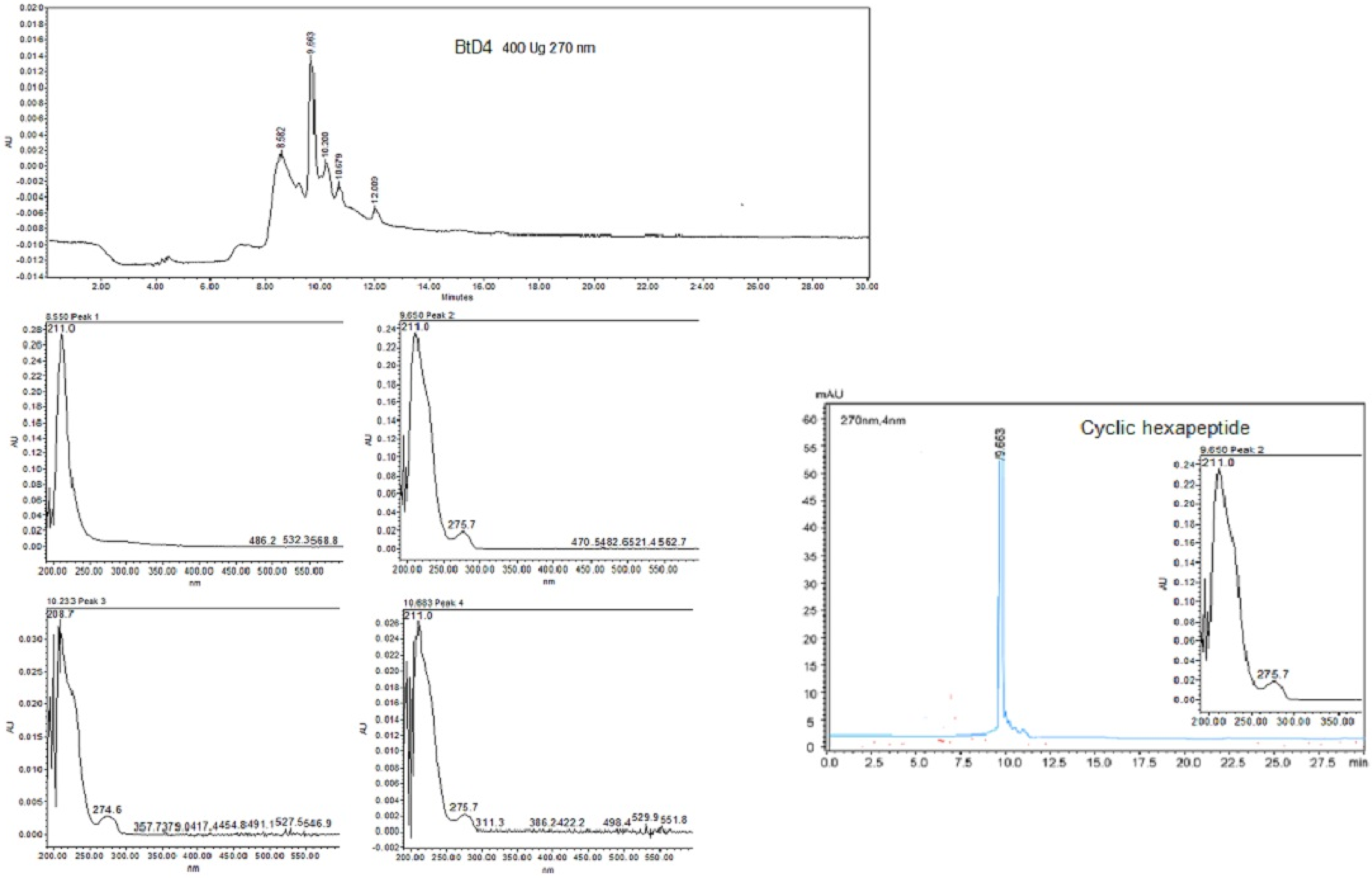

Figure S3. Spectrum UV and HPLC of cyclic hexapeptide

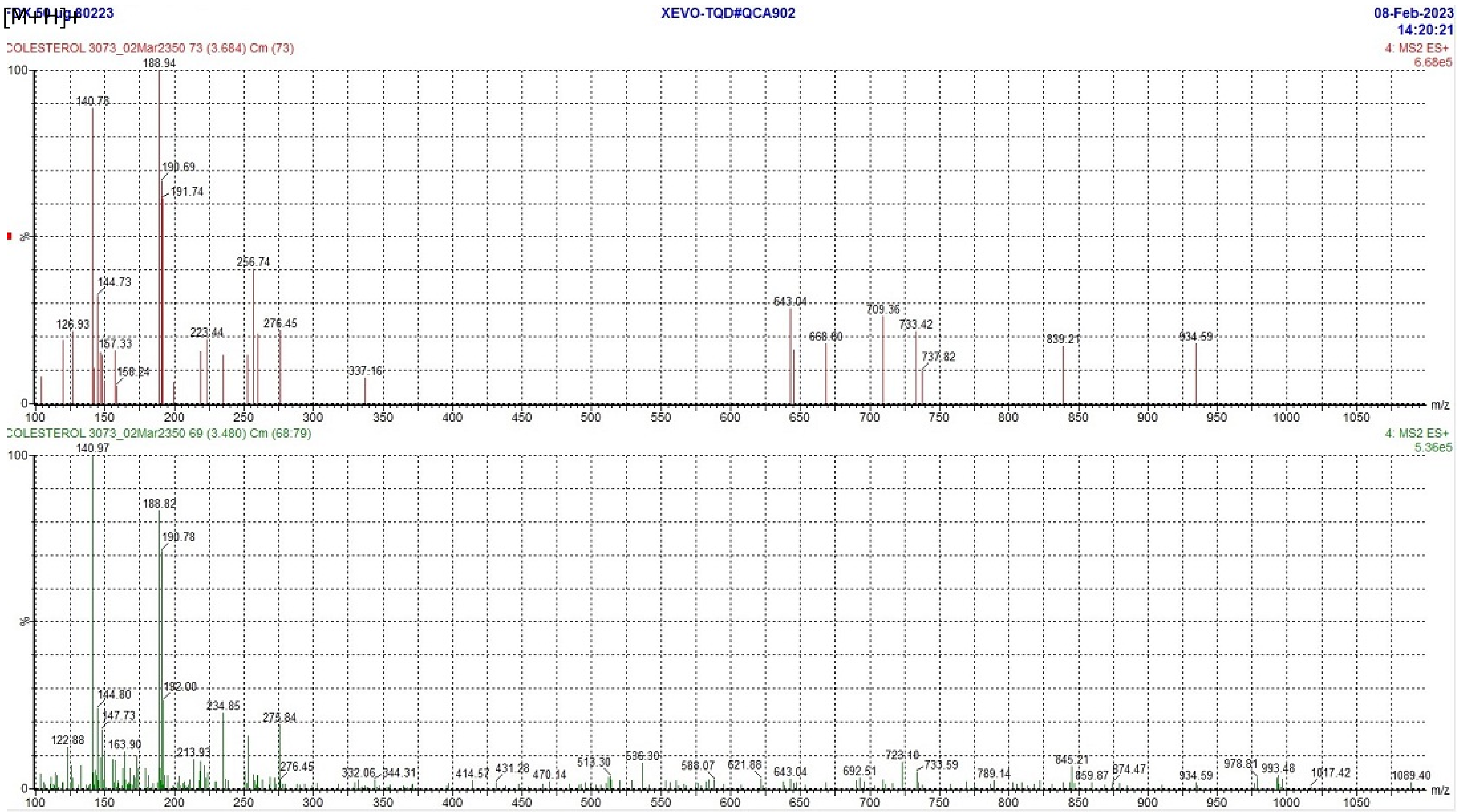

m/z= 934.59 $[M+H]^+$

Figure S4. Mass spectrum (MS) cyclic hexapeptide

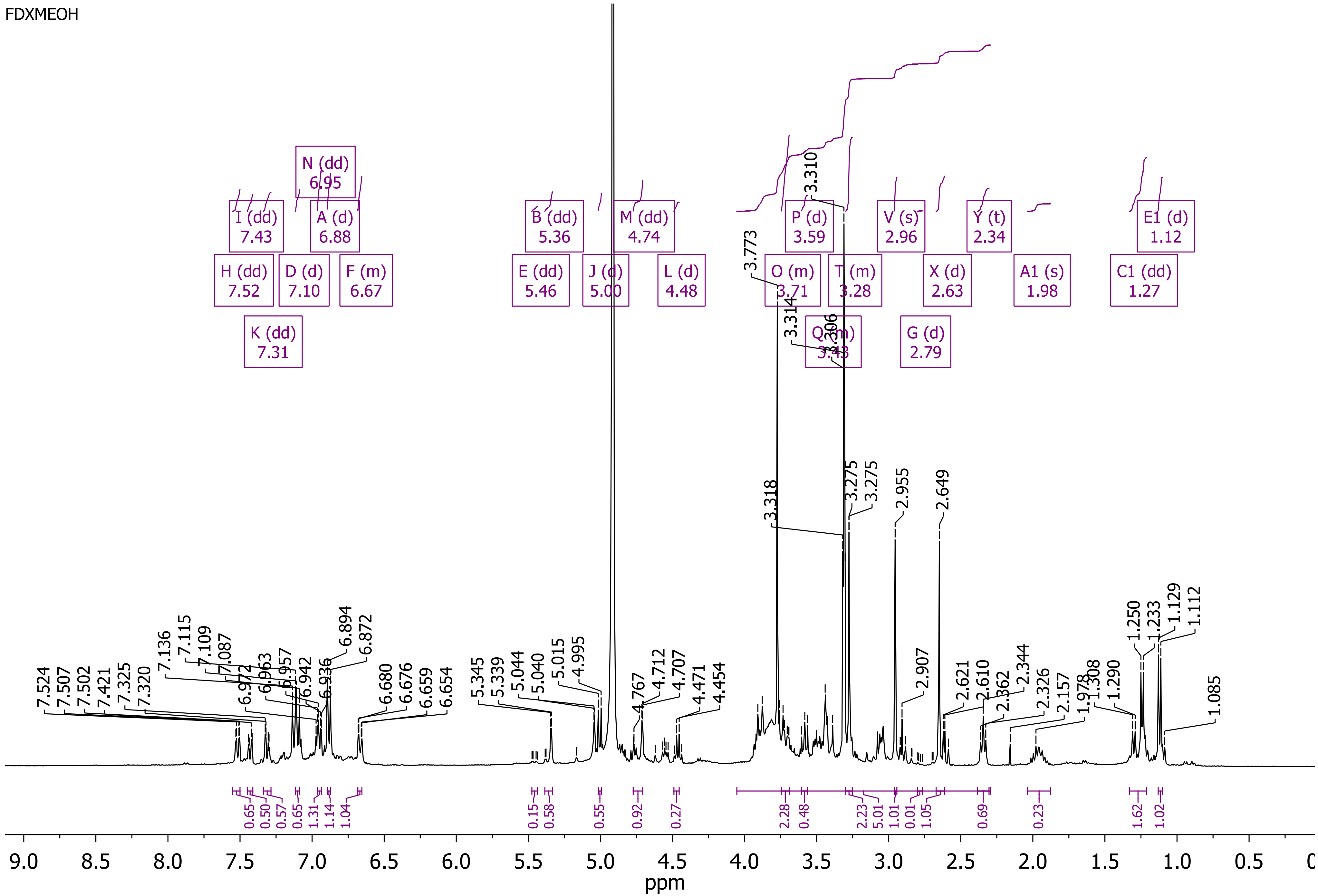

Figure S5. ^{1}H NMR (CD_3OD, 500 MHz) Rubiayunnanesis H

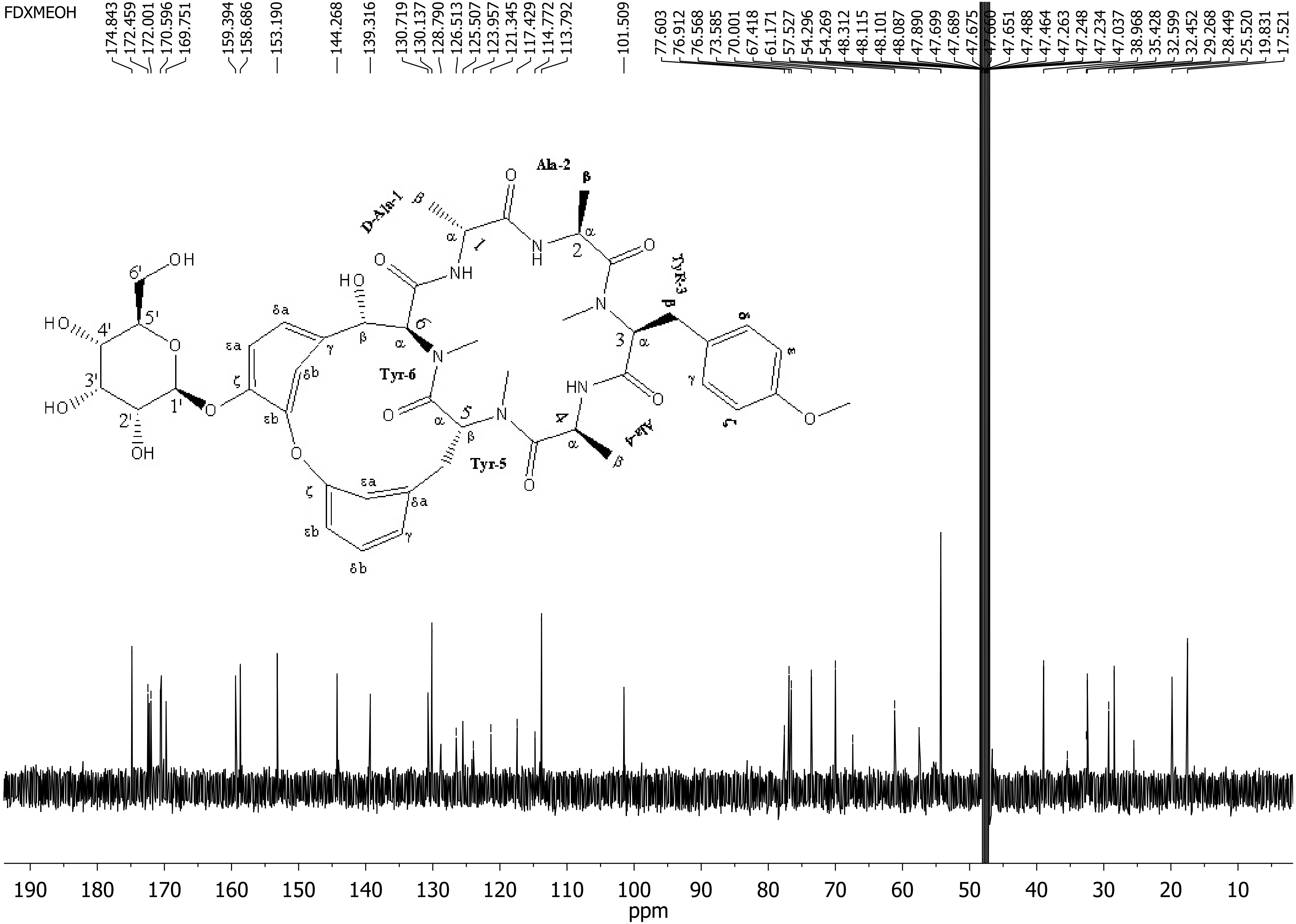

Figure S6. ^{1}H NMR (CD$_3$OD, 500 MHz) Rubiayunnanesis H

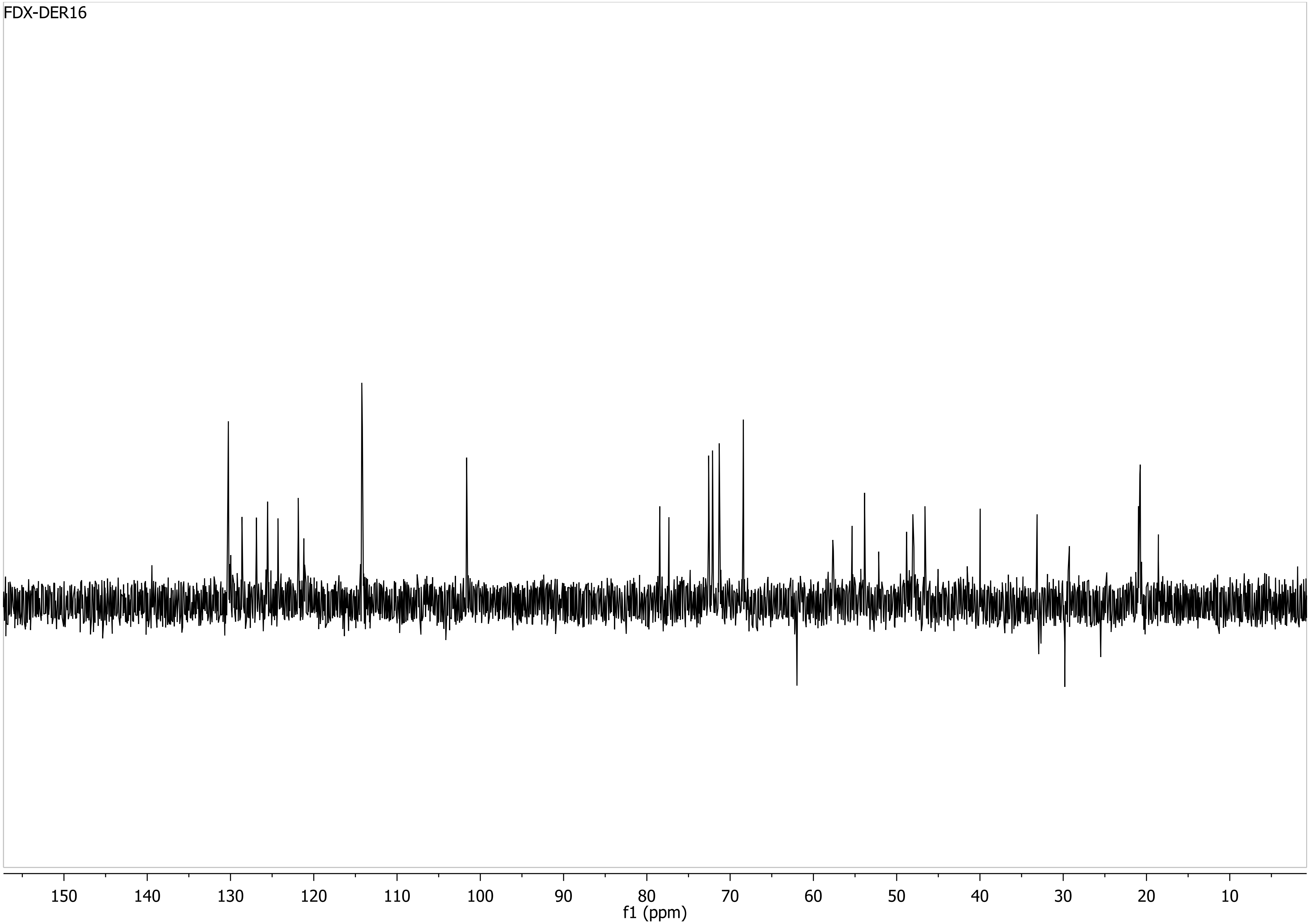

Figure S7. ^{13}C NMR (DEPT) Rubiayunnanesis H

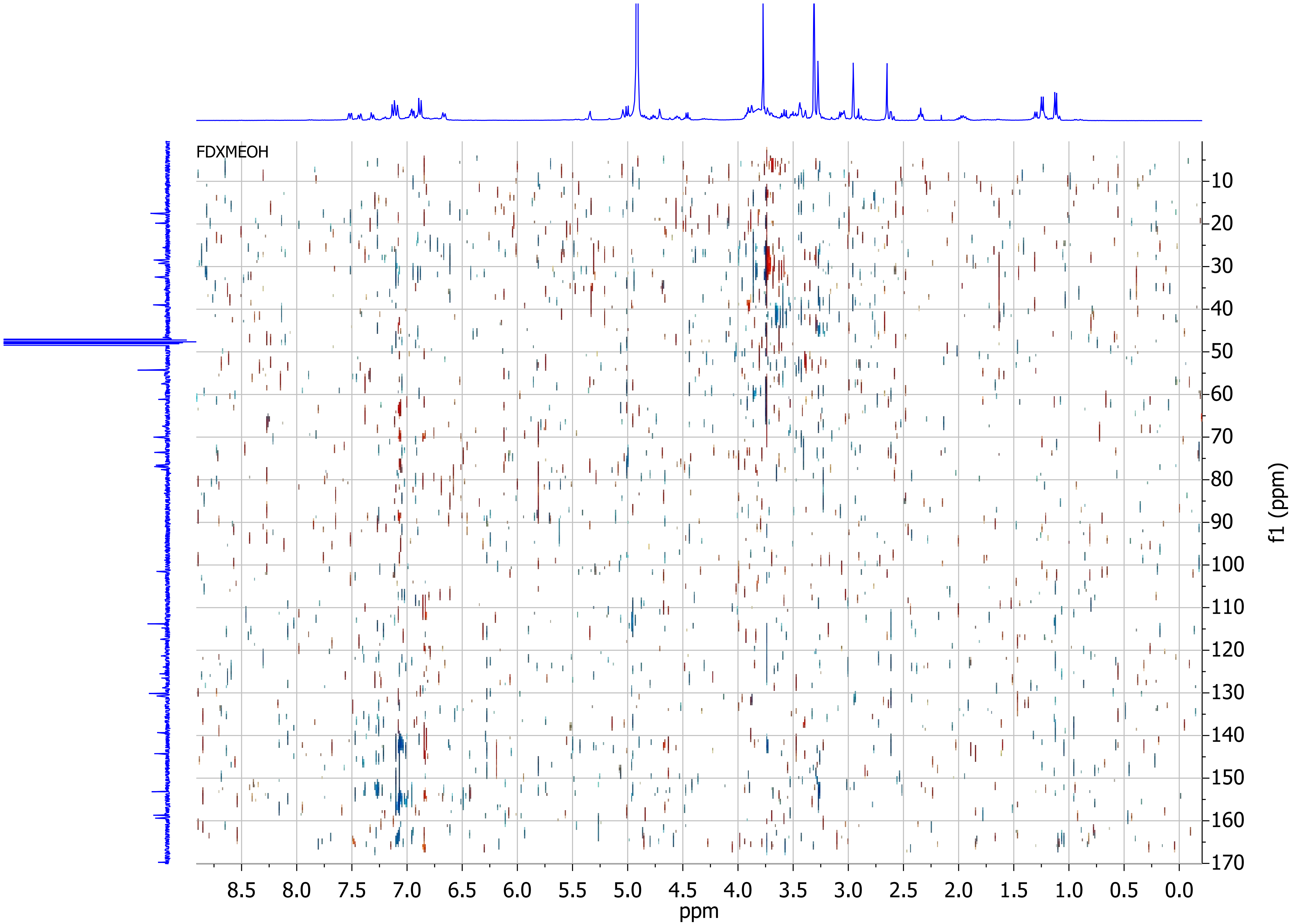

Figure S8. Espectrum HSQC Rubiayunnanesis H

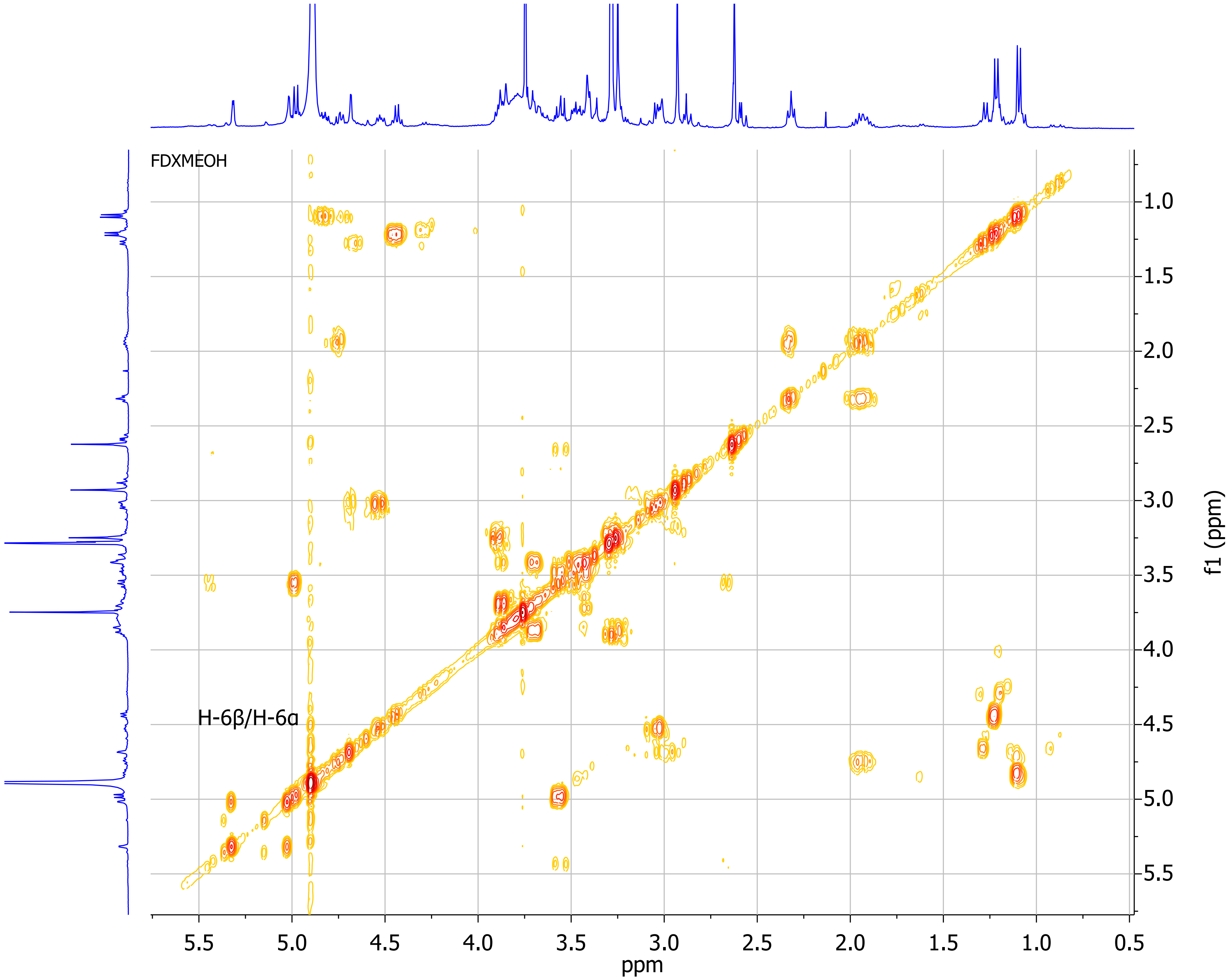

Figure S9. ^{1}H-^{1}H COSY Rubiayunnanesis H
